# Unleash electron transfer in C–H functionalization by mesoporous carbon-supported palladium interstitial catalysts

**DOI:** 10.1093/nsr/nwaa126

**Published:** 2020-06-11

**Authors:** Xiaorui Zhao, Yueqiang Cao, Linlin Duan, Ruoou Yang, Zheng Jiang, Chao Tian, Shangjun Chen, Xuezhi Duan, De Chen, Ying Wan

**Affiliations:** Key Laboratory of Resource Chemistry of Ministry of Education, Shanghai Key Laboratory of Rare Earth Functional Materials, Department of Chemistry, Shanghai Normal University, Shanghai 200234, China; State Key Laboratory of Chemical Engineering, East China University of Science and Technology, Shanghai 200237, China; Key Laboratory of Resource Chemistry of Ministry of Education, Shanghai Key Laboratory of Rare Earth Functional Materials, Department of Chemistry, Shanghai Normal University, Shanghai 200234, China; Shanghai Synchrotron Radiation Facility, Zhangjiang National Lab, Shanghai Advanced Research Institute, Chinese Academy of Sciences, Shanghai 201210, China; Shanghai Synchrotron Radiation Facility, Zhangjiang National Lab, Shanghai Advanced Research Institute, Chinese Academy of Sciences, Shanghai 201210, China; Key Laboratory of Resource Chemistry of Ministry of Education, Shanghai Key Laboratory of Rare Earth Functional Materials, Department of Chemistry, Shanghai Normal University, Shanghai 200234, China; Key Laboratory of Resource Chemistry of Ministry of Education, Shanghai Key Laboratory of Rare Earth Functional Materials, Department of Chemistry, Shanghai Normal University, Shanghai 200234, China; State Key Laboratory of Chemical Engineering, East China University of Science and Technology, Shanghai 200237, China; Department of Chemical Engineering, Norwegian University of Science and Technology, Trondheim N-7491, Norway; Key Laboratory of Resource Chemistry of Ministry of Education, Shanghai Key Laboratory of Rare Earth Functional Materials, Department of Chemistry, Shanghai Normal University, Shanghai 200234, China

**Keywords:** palladium interstitial catalyst, ordered mesoporous carbon, direct bisarylation, multi-site electron transfer, *d*-charge gain

## Abstract

The functionalization of otherwise unreactive C–H bonds adds a new dimension to synthetic chemistry, yielding useful molecules for a range of applications. Arylation has emerged as an increasingly viable strategy for functionalization of heteroarenes which constitute an important class of structural moieties for organic materials. However, direct bisarylation of heteroarenes to enable aryl-heteroaryl-aryl bond formation remains a formidable challenge, due to the strong coordination between heteroatom of N or S and transitional metals. Here we report Pd interstitial nanocatalysts supported on ordered mesoporous carbon as catalysts for a direct and highly efficient bisarylation method for five-membered heteroarenes that allows for green and mild reaction conditions. Notably, in the absence of any base, ligands and phase transfer agents, high activity (turn-over frequency, TOF, up to 107 h^−1^) and selectivity (>99%) for the 2,5-bisarylation of five-membered heteroarenes are achieved in water. A combination of characterization reveals that the remarkable catalytic reactivity here is attributable to the parallel adsorption of heteroarene over Pd clusters, which breaks the barrier to electron transfer in traditional homogenous catalysis and creates dual electrophilic sites for aryl radicals and adsorbate at C2 and C5 positions. The *d*-band filling at Pd sites shows a linear relationship with activation entropy and catalytic activity. The ordered mesopores facilitate the absence of a mass transfer effect. These findings suggest alternative synthesis pathways for the design, synthesis and understanding of a large number of organic chemicals by ordered mesoporous carbon supported palladium catalysts.

## Teaser

The developed ordered mesoporous carbon supported palladium interstitial catalysts demonstrate simultaneous multiple electron transfer and provide alternative paths for heteroarenes synthesis, which overcome the electron transfer restriction of the traditional homogenous catalyst.

## INTRODUCTION

The aryl-heteroaryl-aryl structure is an important structural motif in bioactive molecules, pharmaceuticals and optoelectronic materials [1,2]. The direct arylation of C–H bonds in a heterocyclic core without pre-functionalization prior to carbon–carbon (C–C) coupling, affords a substantially reduced number of reaction steps involved in the synthesis for the elaboration and expansion of the core building unit, and improvement in the sustainability of chemical synthesis [3–6]. For homogenous Pd-catalysed arylation, Pd serves as a single-site centre, involving electron transfer between the C–H bond of heteroarenes and Pd–L (where L denotes the ligand, usually sterically encumbered phosphine ligands) [7,8]. The monoarylation at the C2 or C5 position of pyrroles is obtained by single-site homogenous catalysis [9–14].

Great efforts have been made to achieve bisarylation, but this can only be accomplished by successive monoarylations [15]. For example, the bisarylation of *N*-substituted pyrroles undergoes electrophilic activation in the monoarylation of pyrroles at the C2 position and a following monoarylation. The second monoarylation generally requires adequately soluble proton transfer agents to oxidize the Pd species due to the presence of a phenyl group on *N*-substituted pyrroles [15,16]. The key challenges lie in the variable levels of selectivity for the second arylation at different positions due to the π-excessive property [17], and the limited number of substrates with a sufficient electron density on the pyrrole ring [9,15]. For free pyrrole, only decomposition products are detected [18,19]. There remains an urgent need for direct bisarylation that is more concise, more versatile, or complementary to conventional routes but such has not been documented to the best of our knowledge.

Heterogeneous catalysis behaves differently from its homogenous counterpart which favours a single-site electron transfer. The adsorption of reactants first takes place on the surface active sites of the solid catalyst, and the adsorption configuration and strength have a dominant effect on the activity and selectivity [20,21]. It might be speculated that a heterogeneous catalyst could cause the surface reaction to occur in a different way to the homogenous reaction, and break the barrier to electron transfer on a single site so that direct bisarylation could occur. However, as a result of the presence of a six-electron conjugated π-electron system, pyrrole and thiophene are ready to be adsorbed to transitional metal surfaces, which leads to the most poisonous heterocycles in catalytic reactions, and metal leaching or re-crystallization [22]. Therefore, the first aim is to change the adsorption configuration and energy on metal catalyst surface, which is highly related to the electronic structure of the metal catalyst, according to the Sabatier principle [23]. Previous theoretical calculations suggest that the shifts in the energy of the *d*-states of a Pd(111) surface can be engineered by controlling the metal ligands of the surface atoms and this affects the adsorption energies of nitrogen and oxygen [24]. Promoters in the form of a second metal or non-metal, for example AuPd nanoalloys or C in the surface/subsurface of Pd, can tailor the electronic structure on the surface and thus facilitate favourable catalytic properties [25,26]. The motivation for the use of ordered mesoporous carbon as the support is because porous carbon-supported noble metal catalysts play a prevailing role in the fine chemicals industry and also an indispensable role in new energy technologies, such as biomass conversion, fuel cells and Li-air batteries. Previously we have found that ordered mesopores can inhibit the mass transfer limitation [26].

Herein, for the first time a direct bisarylation of heteroarenes has been demonstrated over a heterogeneous Pd interstitial nanocatalyst supported on ordered mesoporous carbon. The almost identical C2– and C5–H symmetry of pyrroles (free pyrrole and *N*-substituted pyrroles), furan, and thiophene which are adsorbed on the Pd surface means that they can be simultaneously attacked and 2,5-bisarylated products are yielded directly. The reaction is free from the restriction of electron transfer on a single site for a homogenous catalyst. A linear relationship is established between *d* electron increase on Pd sites and activation entropy which is related to the adsorption configuration and strength, and activity. With the most active C,N-modified Pd nanocatalyst supported on ordered mesoporous carbon, high activity (TOF up to 107 h^−1^) and selectivity (>99%) for the 2,5-bisarylation of heteroarenes is achieved in water without using any base, ligands or phase transfer agents. The ordered mesoporous carbon-supported C,N-modified Pd nanocatalyst is stable, retaining almost constant surface Pd concentration, TOF and turn-over number (TON) in the seven successive runs. This strategy paves the way for alternative synthesis paths compared to a homogenous catalyst.

## RESULTS

### Characterization of C,N-Pd/OMC catalyst

**Figure 1.**
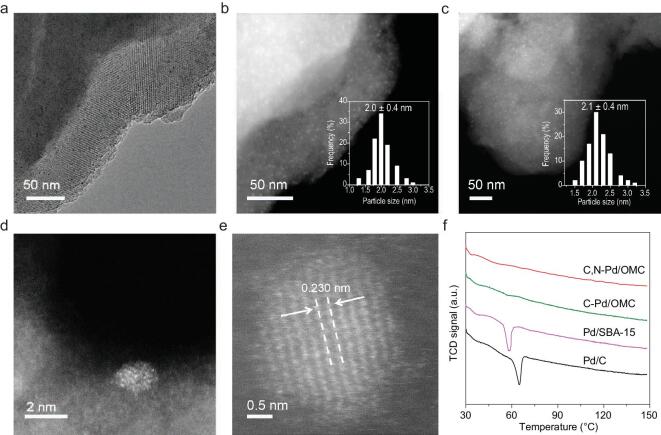
Structure of Pd nanoparticles. (a) Representative TEM image for fresh C,N-Pd/OMC. (b) HAADF-STEM image for fresh C,N-Pd/OMC. (c) HAADF-STEM image for the recycled C,N-Pd/OMC-R7 after seven catalytic runs. (d) HAADF-STEM image of a single particle in (b) with a high magnification. (e) Aberration-corrected HAADF-STEM image of a single particle in (b). (f) TPHD profiles for various solid Pd catalysts: C,N-Pd/OMC, C-Pd/OMC, Pd/SBA-15 and Pd/C. The insets in (b) and (c) are size histograms obtained by measuring at least 200 nanoparticles.

The transmission electron microscopy (TEM) images for C,N-modified Pd supported on ordered mesoporous carbonaceous material (C,N-Pd/OMC) show well-arranged mesopores in large domains and tiny nanoparticles (Fig. 1a). The high-angle annular dark-field scanning transmission electron microscopy (HAADF-STEM) image (Fig. 1b) confirms the presence of monodispersed Pd nanoparticles inside mesochannels with a size of approximately 2.0 nm. The Pd particles have a hemispherical morphology and are partially exposed to the pore space and partially embedded inside the pore walls, providing both accessibility from the substrate and stability by physical confinement (Fig. 1d). N_2_ sorption isotherms show typical type-IV curves, indicative of uniform open mesopores with a mode size of 4.5 nm, a pore volume of 0.44 cm^3^ g^−1^ and a Brunauer-Emmet-Teller (BET) surface area of 355 m^2^ g^−1^ (Supplementary Table 1 and Supplementary Fig. 1). The exposed surface Pd, estimated by pulsing CO chemisorption is 60% and the size of the Pd particles is determined to be 1.8 nm, in good agreement with the TEM results (Supplementary Table 1). Several Pd nanocatalysts supported on porous carriers including C-modified Pd supported on ordered mesoporous carbonaceous material (C-Pd/OMC), and Pd supported on ordered mesoporous silica (Pd/SBA-15) and activated carbon (commercial Pd/C) were also studied. The reference C-Pd/OMC and Pd/SBA-15 show that Pd nanoparticles with average particle size of 2.4 and 3.1 nm, respectively, are well dispersed inside ordered mesopores (Supplementary Fig. 2). But the commercial Pd/C catalyst contains nanoparticles with a wide range of particle sizes (Supplementary Fig. 3). N_2_ sorption isotherms for C-Pd/OMC and Pd/SBA-15 indicate typical uniform mesopores with ranges of pores sizes with modes of 7.8 and 9.0 nm, respectively, and for Pd/C they indicate random mesopores (Supplementary Fig. 4). The pore volumes are larger than 0.4 cm^3^ g^−1^ and the BET surface areas are higher than 300 m^2^ g^−1^ for all catalysts studied (Supplementary Table 1).

An aberration-corrected HAADF-STEM image reveals a slight expansion in the lattice fringe of (111) compared to *fcc* Pd (Fig. 1e). It has been reported that there is a lattice shrinkage for nanosize Pd so that the lattice expansion is proof of a non-metal modification of the surface and subsurface of Pd nanoparticles in C,N-Pd/OMC [27,28]. To further confirm the presence of interstitial C in Pd, temperature-programmed hydride decomposition (TPHD) was carried out which has been proved feasible to detect the subsurface *β*-Pd-H phase with H situating in the octahedral sites of the Pd (Fig. 1f). The evolution of H_2_ at approximately 60°C in the TPHD profiles represents the decomposition of *β*-Pd-H, which is easily formed in supported Pd nanocatalysts upon reduction in hydrogen [29]. Once interstitial carbon is doped in the Pd lattice, the formation of *β*-hydride is inhibited due to the fact that subsurface carbon competes for the same octahedral interstices. The disappearance of this negative H_2_ evolution peak gives evidence for the presence of subsurface carbon in Pd in both C-Pd/OMC and C,N-Pd/OMC catalysts. In contrast, Pd/SBA-15 and commercial Pd/C have no interstitial carbon atoms [27]. The C atoms may originate from the thermal release of small molecules such as CH_4_ during the heat treatment of the carbonaceous carrier (Supplementary Fig. 5). The adsorption of CH_4_ on the Pd surface leads to its decomposition, C deposition and C transfer into the subsurface driven by heat [30,31].

**Figure 2.**
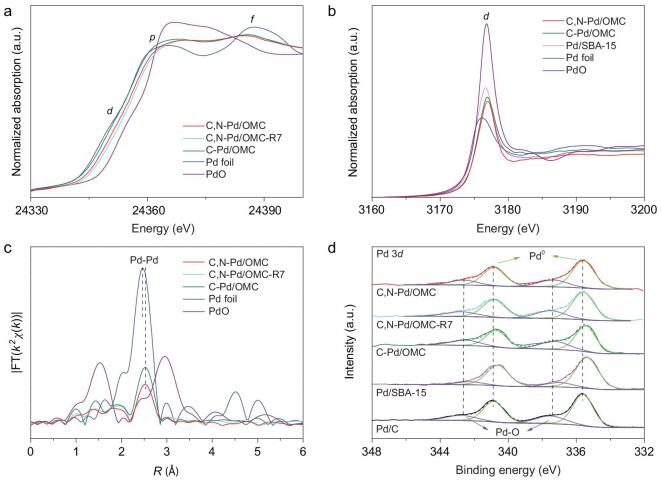
Compositions and electronic properties. (a) XANES spectra of the Pd *K*-edge of C,N-Pd/OMC, C,N-Pd/OMC-R7 and C-Pd/OMC catalysts and reference samples (Pd foil and PdO). The Pd XANES spectrum at the *K*-edge of the Pd foil exhibited a pronounced white line due to the unfilled Pd *d* band, and the spectrum of PdO exhibited an even more obvious white line that shifted to higher threshold energy because of the higher oxidation state of Pd in PdO. The hybridization-mediated 1*s*→4*d, dp (d)* absorption transition was in the pre-edge region. The near-edge spectrum showed two resonance peaks that are due to the 1*s*→5*p* (*p*) and 1*s*→4*f* (*f*) transitions. The shape of the second absorption edge reflected the extent of the 4*d*-5*p* hybridization. This feature was insignificant in the spectrum of the PdO reference. (b) XANES spectra of the Pd *L*_3_-edge of C,N-Pd/OMC, C-Pd/OMC and Pd/SBA-15 catalysts and reference samples (Pd foil and PdO). The white line intensity has been measured with respect to the absorption intensity from approximately 3 below to 15 eV above the threshold. (c) The *k*^2^-weighted and Fourier transformed magnitudes of the extended X-ray absorption fine structure (EXAFS) spectra of the Pd *K*-edge. The Fourier transforms were not corrected for phase shifts. (d) X-ray photoelectron spectroscopy (XPS) of the 3*d* level of Pd for the studied solid catalysts. The Pd binding energies were fitted by peak fitting programs.

The electronic properties for the Pd catalysts with the surface or subsurface carbon were investigated. The pre-edge line in the Pd *K*-edge X-ray absorption near edge structure (XANES) spectrum of the C-Pd/OMC catalyst is similar to the reference spectrum of the Pd foil, indicating that the Pd is mainly in its metallic state (Fig. 2a). But the three essential features arising from *d*-, 5*p*- and 4*f*-orbitals, respectively appear different. In particular, the *d* peak has disappeared and the *p* peak is broadened, indicating that the top of the 4*d* band has fallen in energy [32]. As a result, the 4*d* band is filled. This phenomenon suggests some non-*d* charge depletion at the Pd site. The depletion of *p* charge in Pd is also evident from the increase in the absorption intensity for the *p* peak. The broadening of the *p* peak and its 1 eV shift to a higher energy are in good agreement with those in palladium carbide [33,34]. Therefore, an electronic perturbation of the Pd atoms occurs possibly going from a Pd-like environment in the monometallic structure to PdC*_x_*. The 1*s*→4*f* peak is shifted to lower energies (1.5 eV), accompanied by a significant reduction in intensity, possibly due to the increase in the atomic distance and 4*d*-5*p* hybridization [35]. Similar phenomena have been observed for local distortions of the Pd lattice by interstitial C [33]. The XANES spectrum for C,N-Pd/OMC is quite similar to that for C-Pd/OMC, but a slight shift of the white line to higher energies indicates the presence of Pd–O species, and a possible increasing degree of *d*-band filling.

To shed light on the electronic effect of C and N on Pd, XANES measurements were performed at the Pd *L*-edge to probe unoccupied valence states (Fig. 2b). It has been reported that cluster samples show a high white line intensity which is attributed to the formation of a band gap and localization of empty state wave functions in small clusters [36]. As expected, the Pd/SBA-15 catalyst shows a predominantly enhanced white line intensity compared to a Pd foil, and a shift to a higher energy. To eliminate the size effect, the electronic properties for C, N-Pd/OMC and C-Pd/OMC are thereby compared with Pd/SBA-15 with similar particle sizes. The edge shifts to a higher energy, and the white line peak decreases in both catalysts, demonstrating the formation of interstitial carbon in the Pd lattice, and the depletion of empty *d* states [33,37]. The intensity difference for the *L*_3_ white line between C-Pd/OMC or C,N-Pd/OMC and Pd/SBA-15 was then used to calculate the occupancy of the *d* valence band [38]. It is apparent from the correlations that the *d* band gets filled when Pd is doped by carbon and carbon/nitrogen. The *d* electron gain is estimated to be 0.0693 and 0.1649 for C-Pd/OMC and C,N-Pd/OMC interstitial catalyst, respectively.

The dominant peak in the *k*^2^-weighted and Fourier-transformed (FT) Pd *K*-edge EXAFS spectra for C-Pd/OMC and C,N-Pd/OMC corresponds to the closest Pd–Pd atomic pairs (Fig. 2c). The dramatically reduced intensity compared to that of a Pd foil is due to the coordination-unsaturated Pd sites. The Pd–Pd coordination number (CN) is reduced to 5–6 for these two catalysts (Supplementary Table 2). In addition, the Pd–Pd distance (*R*) increases to 2.77–2.78 Å from 2.75 Å. It has been established that the metal–metal bond length decreases as nanoparticle size decreases, due to surface strain [39]. An earlier study found that the Pd–Pd distance shrinks to 2.71 Å owing to the reduction of the Pd nanoparticle size to approximately 2 nm [40]. Therefore, the increase in *R*_Pd–Pd_ may be a signal to the lattice expansion originated from the carbon doping into Pd lattice [28], in accordance with the aberration-corrected HAADF-STEM results. None of the characteristic features for Pd–O–Pd distance of Pd oxide at approximately 2.9 Å are detected for the present catalysts. The formation of an oxide (Pd–O–Pd) can be excluded therefore, but a surface Pd–O bond can be formed. The signals at approximately 2.0 Å (phase uncorrected) for C-Pd/OMC and C,N-Pd/OMC differ from the reference foil. The peaks in this area were investigated due to the possibility of metal–carbon (Pd–C) pairs. The EXAFS data were then fitted with three theoretical contributions: one originating from Pd in a metallic environment (Pd–Pd), the second corresponding to Pd–O(N) since the Pd–O and Pd–N coordination cannot be easily identified, and a third attributed to Pd–C. Pd–C coordination with a CN of approximately 1.4 and a bond distance of 2.19 Å is observed for C-Pd/OMC. This coordination is similar in C,N-Pd/OMC. The increase of intensity and CN for Pd–O(N) in C,N-Pd/OMC compared to C-Pd/OMC is possibly related to Pd–N coordination in the former.

Elemental analysis shows that the catalyst contains nitrogen with a molar ratio of N:Pd of 4.1. The XPS at the N 1*s* level for the as-made OMC displays a peak at 402.5 eV which is assigned to the nitrogen in a quaternary ammonium group [41], indicating the introduction of N-containing groups into the mesoporous carrier (Supplementary Fig. 6). Upon heating at 275°C, a dramatic change occurs. A major peak appears at 399.4 eV, corresponding to the N–H species, suggesting the transformation of quaternary ammonium groups to N–H species. The impregnation with Pd salt leads to a shift of this peak to a higher binding energy, indicative of the Pd–N coordination, in good agreement with metallic nanoparticles supported on N-containing carriers including carbon nanotubes and graphene [42,43]. After reduction, the binding energy for the N–H species is maintained. The typical doublets for the Pd 3*d* XPS spectra can be deconvoluted (Fig. 2d), and the major peaks for Pd^0^ and the minor peaks for Pd–O can be fitted. Compared to the reference catalyst of C-Pd/OMC which has no elemental N in the carrier and Pd/SBA-15 which has a very weak interaction with Pd, the Pd 3*d* core levels of the C,N-Pd/OMC show a shift towards a higher binding energy. Since the particle sizes for C-Pd/OMC and C,N-Pd/OMC are close, the shift in binding energy may be attributed to an increase of *d* occupancy in C,N-Pd/OMC due to the presence of N atoms surrounding Pd, which would move the Fermi level closer to the top of the *d* band [44].

### Direct bisarylation of *N*-methylpyrrole in water over C,N-Pd/OMC

For the bisarylation reaction, water and diaryliodonium salts (diphenyliodonium trifluoromethanesulfonate, Ph_2_IOTf) were chosen as the solvent and arylating reagent. No other additives, including phosphine ligands, base or phase transfer agents were used. Preliminary experiments were performed to exclude the diffusion mass transfer limitations. By varying the stirring speeds from 400 to 1 000 rpm, the results showed that a speed of 800 rpm enabled the reaction to perform without diffusion limitations. A Madon-Boudart (MB) test was then carried out using a series of catalysts with different surface concentrations of metal but similar dispersions [45]. C,N-Pd/OMC catalysts with Pd loadings of 0.51, 0.98 and 1.50 wt% showed similar N_2_ sorption isotherm curves and chemisorption results (Supplementary Table 1 and Supplementary Fig. 1). A linear relationship indicated the absence of a mass transfer effect under the present reaction conditions (Supplementary Fig. 7). The easy accessibility of metal nanoparticles intercalated in the framework was related to the mesopores being large enough.

**Figure 3.**
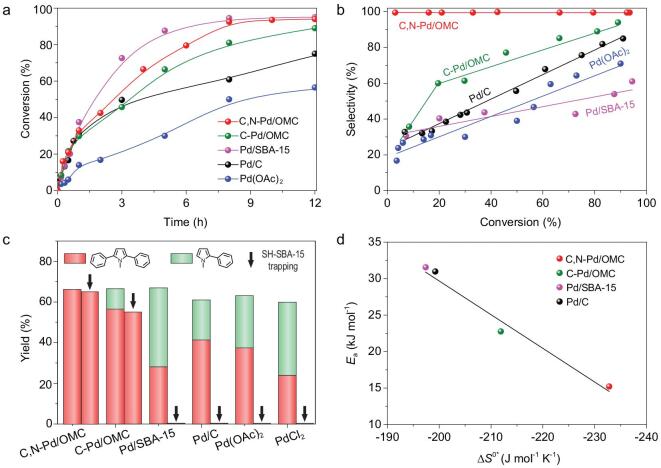
Kinetics study. (a) Conversion plots for the Pd-catalysed arylation of *N*-methylpyrrole with reaction time. (b) Compilation of the selectivity to *N*-methyl-2,5-diphenylpyrrole as a function of conversion. Reaction conditions: 1 mol% of Pd catalyst; 0.2 mmol of *N*-methylpyrrole; 0.4 mmol of Ph_2_IOTf; 2 mL of water; 60°C; atmospheric pressure; in air. (c) Comparison of the yields of the bisarylated and monoarylated *N*-methylpyrroles between the pure reaction and reaction in the presence of a solid SH-SBA-15 trapping agent over various Pd catalysts (S:Pd = 35 in molar ratio) at the conversion of approximately 60%. (d) Relationship between *E*_a_ and Δ*S*^0*^ for the direct bisarylation of *N*-methylpyrrole to *N*-methyl-2,5-diphenylpyrrole on various solid Pd catalysts.

The C,N-Pd/OMC interstitial catalyst exhibits a high activity (TOF value of 107 h^−1^) and selectivity (>99%) in the bisarylation of *N*-methylpyrrole at 60°C in water (Fig. 3). *N*-methyl-2,5-diphenylpyrrole is the only detected product during the whole reaction (Supplementary Fig. 8). With a scaled-up reaction, the reaction is completed after stirring for 8 h. The isolated *N*-methyl-2,5-diphenylpyrrole yield remains 90% and *N*-methyl-2-phenylpyrrole cannot be obtained. Even for free pyrrole, similar results are obtained with the exclusive product of 2,5-diphenylpyrrole with the conversion of 92% at 8 h. Although we cannot directly compare the TOF value with literature values (in most cases, conversion plots were not provided), this value is estimated to be much higher than that in homogenous catalysis for monoarylation (5 mol% Pd, 24 h for ∼60% conversion of *N*-methylpyrrole in *N*-methyl pyrrolidone [46]) due to the lower amount of Pd used and a short reaction time with a high conversion. The *N*-methylpyrrole concentration was tested for the reaction order. The approximate constant of the reaction rates along with the given *N*-methylpyrrole concentration ranges would suggest that the reaction is pseudo zero order (Supplementary Fig. 9). From the Arrhenius plots in Supplementary Fig. 10, the apparent activation energy (*E*_a_) and the activation entropy (Δ*S*^0*^) are determined to be 15.21 kJ mol^−1^ and −232.8 J mol^−1^ K^−1^, respectively, according to transition state theory. The low *E*_a_ value is reflected by the high negative Δ*S*^0*^, indicating a significant change in the mobility of the reactant going from the initial to the activated state and maybe related to the radical-involved reaction [47,48].

In this case, successive monoarylation and direct bisarylation are the two possible routes for the production of the bisarylated chemical. However, a negligible amount of monoarylated product is detected during the reaction. One may assume the addition of two equivalents (equiv.) of Ph_2_IOTf results in successive monoarylation similar to what is observed for homogenous catalysis. If 1 equiv. of Ph_2_IOTf was added, the selectivity for *N*-methyl-2,5-diphenylpyrrole remained the same, but the conversion of *N*-methylpyrrole was reduced by about half. This phenomenon is completely different from the homogenous Pd-catalysed successive arylation, in which a decrease in the amount of arylation agent yielded a monoarylation product with almost no bisarylated product produced [9]. Another doubt is that the monoarylation product of *N*-methyl-2-phenylpyrrole may undergo an extremely rapid conversion and then serve as the reactant. A bisarylation product could be produced, but the initial reaction rate (17 mmol mmol_Pd_^−1^ h^−1^) was much lower than that for *N*-methylpyrrole (64 mmol mmol_Pd_^−1^ h^−1^). The monoarylation product should not serve as the intermediate. In addition, a competitive reaction between *N*-methylpyrrole and monoarylated chemical was carried out. The addition of *N*-methyl-2-phenylpyrrole to the reaction batch did not have a distinct effect on the conversion of *N*-methylpyrrole (92% of conversion at 8 h), but the conversion of *N*-methyl-2-phenylpyrrole was as low as 8%, reflecting the inhibition of the arylation of *N*-methyl-2-phenylpyrrole in the presence of pyrrole. As a result, the possibility of the occurrence of successive monoarylation over C,N-Pd/OMC can be ruled out.

The leaching of Pd from solid catalysts has long been argued to contribute to the catalytic activity. Pd/C acts as a metal reservoir of active Pd species [49,50]. Pd leached into solution from the Pd/C nanoparticles and some aggregates to form Pd*_n_*^0^ clusters, both of which can catalyse the C–H functionalization and C–C bond coupling reactions, and may be redeposited on the support after the reaction [49]. To exclude the effect of leaching species, a solid trapping test was carried out [27]. Thiol-functionalized mesoporous silica (SH-SBA-15) was added to the synthesis mix with a SH:Pd molar ratio of 35. Once the soluble palladium is released from the catalyst, the solid trapping agent easily and efficiently captures it, inhibiting the following agglomeration of Pd*_n_*^0^ clusters, and therefore quenching the reaction. The results show no significant difference in the conversion of *N*-methylpyrrole and the yield of bisarylated product over C,N-Pd/OMC in the presence of SH-SBA-15 compared to the reaction without it, confirming negligible Pd leaching into solution (Fig. 3c). A hot filtration experiment was also carried out (Supplementary Fig. 11) in which the C,N-Pd/OMC catalyst was hot filtered at 60°C after 30 min and no additional conversion was observed, even when fresh substrate and diphenyliodonium were added to the filtrate. Recycling tests of the C,N-Pd/OMC interstitial catalyst were also performed (Supplementary Fig. 12). The initial reaction rate, which is dominated by the overall active sites, the selectivity to *N*-methyl-2,5-diphenylpyrrole, as well as the TON remained almost constant in seven successive runs. After each run, the filtrate was collected and the mixture examined by inductively coupled plasma-atomic emission spectrometry (ICP-AES), and the concentration of metal ions was under the detection limit. These results confirm that there was no leaching of Pd into solution. After seven catalysis runs, the XPS spectrum at the Pd 3*d* level for C,N-Pd/OMC is analogous to that of the fresh catalyst (Supplementary Fig. 13) with a similar surface Pd concentration. A slight increase in the Pd–O signal may be attributed to adsorbed organic compounds. A slight shift of white line to higher energies in the XANES spectrum for the reused catalyst compared to that for the fresh catalyst may be also due to residual organic compounds on the Pd surface (Fig. 2a). The reused C,N-Pd/OMC catalyst shows a quite similar FT-EXAFS spectrum to the fresh one, further confirming the almost unchanged chemical environment of Pd during the reaction (Fig. 2c). Characterization by CO chemisorption and TEM images (Supplementary Table 1 and Fig. 1c) show similar results with the fresh catalyst, confirming the stability in the absence of aggregation for the Pd clusters even in the presence of a strong adsorbate. These results confirm these studies of surface catalysis and the almost unchanged surface Pd concentration. The XPS spectrum for the used C-Pd/OMC catalyst is similar to the fresh one, but the surface Pd concentration is reduced, implying 8.5% Pd is dissolved in the solution from the surface and the remaining surface Pd is stable.

### Monoarylation vs. direct bisarylation of *N*-methylpyrrole in water

Compared to C,N-Pd/OMC, the homogenous catalyst Pd(OAc)_2_ showed a much lower reaction rate (*r*_0_ = 12.6 mmol mmol_Pd_^−1^ h^−1^) in the arylation of *N*-methylpyrrole (Fig. 3). It should be mentioned that the conversion continues to increase even after 35 h, implying that the aggregation to inactive Pd black is inhibited to some extent. The yield of the monoarylated product *N*-methyl-2-phenylpyrrole shows a rapid increase to a maximum, followed by an extremely slight decrease, and the bisarylated product of *N*-methyl-2,5-diphenylpyrrole has an increasing yield (Fig. 3b and Supplementary Fig. 14). In a separate experiment, activated carbon was added to the Pd(OAc)_2_-catalysed reaction mix after 1 h. TEM images clearly showed Pd particles on the filtered activated carbon (Supplementary Fig. 15). This observation is in good agreement with published results that Pd(OAc)_2_ easily undergoes agglomeration into Pd*_n_*^0^ clusters and is finally deactivated by the formation of Pd black [51]. To study the product catalysed by Pd ions and avoid the rapid agglomeration of Pd, an extremely low concentration of Pd(OAc)_2_ was also used in the arylation of *N*-methylpyrrole (Supplementary Fig. 16). Initially, both the reaction rate (*r*_0_ = 192 mmol mmol_Pd_^−1^ h^−1^) and the selectivity (>99%) to the monoarylation product of *N*-methyl-2-phenylpyrrole were extremely high. For the production of bisarylated pyrrole, kinetic studies indicate a significant induction period and a sigmoidal reaction profile. Once the bisarylated product appears, the reaction rate is significantly reduced. These phenomena imply the generation of new active sites during the reaction which are possibly insoluble Pd*_n_*^0^ clusters from the initial Pd ions. In addition, a homogenous PdCl_2_ catalyst was used. Both monoarylated and bisarylated products were obtained at a conversion of approximately 60%, similar to the results using Pd(OAc)_2_. When SH-SBA-15 was added to the homogenous catalytic reaction using either Pd(OAc)_2_ or PdCl_2_, no products were detected (Fig. 3c). We therefore assume that homogenous Pd(OAc)_2_ catalyses the monoarylation of *N*-methylpyrrole at the very beginning and the initial reaction rate is exclusively due to Pd^2+^ in dilute Pd(OAc)_2_. Pd aggregation occurs especially when there is a high Pd(OAc)_2_ concentration. Accompanied by the formation of Pd*_n_*^0^ clusters after the induction period, *N*-methyl-2,5-diphenylpyrrole is produced. The continuously increasing selectivity to the bisarylated product in the Pd(OAc)_2_-catalysed system is due to the rapid formation of Pd*_n_*^0^ clusters as active centres. Once all Pd ions aggregate into Pd*_n_*^0^ clusters, which can transform *N*-methylpyrrole to *N*-methyl-2,5-diphenylpyrrole, the reaction rate is low.

The reference Pd/C, Pd/SBA-15 and C-Pd/OMC catalysts were also investigated. The Pd nanoparticle sizes in the latter two catalysts are similar to that of C,N-Pd/OMC, a particle size effect for the catalysis can therefore be confidently excluded. In the case of the commercial Pd/C catalyst, the reaction rate is higher than that for Pd(OAc)_2_ but the selectivity plot along with the conversion is similar. Monoarylated and bisarylated products are produced and the selectivity for the latter increases (Fig. 3b). The solid trapping agent SH-SBA-15 quenches the reaction (Fig. 3c), confirming the leaching of Pd from the Pd/C and the subsequent formation of Pd*_n_*^0^ clusters that serve as active centres, in good agreement with the literature [49]. The TEM images for the separate solids (catalyst and trapping agent) show hexagonally arranged mesopore domains which belong to SBA-15 (Supplementary Fig. 17). Although Pd nanoparticles cannot be clearly seen, the energy dispersive X-Ray spectroscopy (EDS) pattern shows the presence of Pd with a concentration of 0.72 wt% in these ordered mesostructure domains. This value is close to the theoretical one (0.75 wt%), suggesting almost complete leaching of Pd from Pd/C during the reaction. The increasing reaction rate over Pd/C compared to homogenous Pd(OAc)_2_ implies a mild release of Pd ions, stabilization of active Pd*_n_*^0^ clusters by the carbon support and inhibition of aggregation into extremely large Pd clusters in a short time. The reused catalyst has a very low activity. TEM images for the used Pd/C catalyst reveal the presence of large particles, which is evidence for the dissolution-redeposition of Pd and the formation of Pd black on activated carbon (Supplementary Fig. 3) [51].

Compared to Pd/C, Pd/SBA-15 has a similar initial reaction rate, but it is much higher after 1 h, and the selectivity for the bisarylated product is lower at a high conversion. Similarly, SH-SBA-15 acts as an effective soluble Pd scavenger released from Pd/SBA-15 which also serves as the metal reservoir. These phenomena can be explained by the slower release rate of Pd ions from mesoporous silica SBA-15, and the dilute solution inhibits the rapid formation of clusters. As a consequence, both the reaction rate during the whole reaction and the maximum yield for the monoarylated product is the highest among all studied catalysts.

At a conversion below 10%, the rate and selectivity to the bisarylated product over C-Pd/OMC are similar to those over Pd/C and Pd/SBA-15 (Fig. 3b) with the by-product of monoarylated product. As the reaction proceeds, the selectivity for the bisarylated product dramatically increases. The maximum yield of the monoarylated product is the lowest among all the studied catalysts. Note that the presence of SH-SBA-15 only quenches monoarylation while the yield for the bisarylated product remains unchanged (Fig. 3c). These phenomena indicate that there are two kinds of active centres in C-Pd/OMC: a small number of leached Pd species and surface Pd; monoarylation is exclusively catalysed by single-site homogenous leached Pd. The presence of interstitial carbon in the Pd lattice can improve the stability of surface Pd to some extent, but leaching still occurs in this N-free catalyst. The presence of leaching Pd is the reason for the similar activity for conversion of *N*-methyl pyrrole over C-Pd/OMC but lower selectivity to bisarylated product compared with those over C,N-Pd/OMC.

It should be mentioned that an extremely slow transformation from *N*-methyl-2-phenylpyrrole to *N*-methyl-2,5-diphenylpyrrole occurs over all these catalysts, in good agreement with the above result that the reaction rate for the former is extremely low in the presence of *N*-methylpyrrole over surface Pd.

We roughly separate the reaction rate into monoarylation and direct bisarylation rates catalysed by soluble Pd and Pd*_n_*^0^ clusters, respectively. The Pd*_n_*^0^ clusters are assumed to adsorb on the porous carriers once they are formed. The rapid formation of Pd black is hindered to some extent. The activation energy and activation entropy were estimated considering the initial reaction rate over the Pd*_n_*^0^ clusters. The *E*_a_ and Δ*S*^0*^ values are comparable to those over C,N-Pd/OMC. Changes in activation energy for the Pd catalysts are offset by a change in the activation entropy, which is the so-called ‘compensation effect’ [52]. This effect has been explained by relating the entropy of transition to a change in the energy levels of the transition state. The freedom of the transition system increases, due to loosened bonds with the surface and adsorbed molecules, as the energy of the system increases [53]. Therefore, the activation entropy changes must be inherent to the electronic properties of the solid [26].

## DISCUSSION

### Surface *d* charge gain at Pd sites as the descriptor for activation entropy and catalytic activity

**Figure 4.**
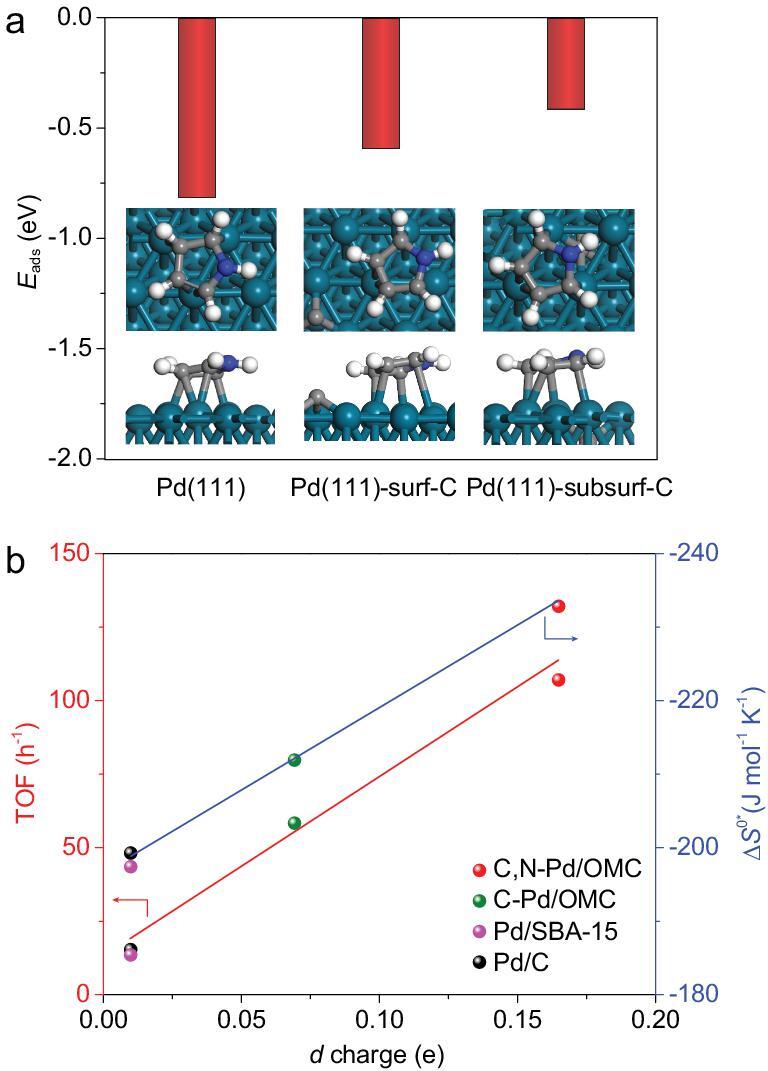
DFT calculations and descriptor for the catalytic performance. (a) The adsorption energy of pyrrole and the favourite configuration over Pd(111), surface-C-modified Pd(111) and subsurface-C-modified Pd(111) by DFT calculations. (b) Relationship between the *d-*charge gain at Pd sites and TOF (red line) of the Pd catalysts supported on various carriers, and the entropy of activation for the direct bisarylation of *N*-methylpyrrole (Δ*S*^0*^, blue line).

The five-membered heteroarenes contain delocalized electron pairs, in which the heteroatom has at least one pair of non-bonding valence shell electrons. The adsorption of pyrrole on clean Pd(111) surfaces was studied by density functional theory (DFT) calculations. The pyrrole molecule adopts an almost flat geometry without C–H or N–H bond cleavage (Fig. 4a). Similar adsorption configuration on Al(100) and Ru(0001) surface has been reported [54,55]. The simultaneous activation of the C–H bonds at the C2/C5 sites of pyrrole is evidenced by the elongation of these bonds compared to the isolated pyrrole molecule (C–H bond length of 1.085 Å). The adsorption energy is relatively negative, suggesting strong adsorption on Pd(111). The strong adsorption may poison the surface or trap the surface atoms from nanoparticles into solution. The effect of the presence of surface and subsurface C in Pd(111) on the adsorption energy for pyrrole was further investigated. The adsorption energy is predominantly reduced with the elongation of the C2/C5–H bonds. The calculations on pyrrole adsorption on the co-existence of C and N in Pd(111) deserves further investigation.

To gain insight into the reaction mechanism, radical scavengers including 2,2,6,6-tetramethyl-1-piperidinyloxy (TEMPO), 1,4-benzoquinone (BQ) and 2,6-di-tert-butyl-4-methylphenol (BHT) were added to the reaction (Supplementary Fig. 18). TEMPO and BQ almost completely quench the reaction. A low conversion of 19% is obtained with BHT possibly due to its low solubility in water so that its low concentration is insufficient to completely trap radicals. Deuteration experiments using D_2_O or CD_3_OD as a deuterium reagent in the absence of diaryliodonium salts under reaction conditions produce undetected deuterated *N*-methylpyrrole, suggesting the inhibition of the C–H bond cleavage and the formation of radical species by pyrrole hydrogen atom abstraction (Supplementary Fig. 19). Since the electrophilic aryl radicals which are generated by Ph_2_IOTf have been proven to be active in arylation by both experiments and calculations between phenyl radical and heteroaromatics, a radical coupling path for the arylation reaction is possible [50,56].

The kinetic isotope effect (KIE) was then studied over C,N-Pd/OMC. The *k*_H_/*k*_D_ value for the bisarylation is estimated to be approximately 1.0, both in parallel and competitive experiments with pyrrole and pyrrole-*d*5 (Supplementary Figs 20 and 21). The slight difference is possibly due to the zero-point energy difference between isotopic isomers [57]. As a result, the C–H bond cleavage may not be involved in the rate-determining step. The surface adsorption/desorption step is possibly the rate-determining step of the catalytic reaction. The change in reaction rate does indeed follow the activation entropy variations (Supplementary Fig. 22).

It has been reported that electron excitation is closely related to either an adsorption or a desorption step, i.e. the promotion of electrons from sub-Fermi levels up to the Fermi surface of the electronic conduction band of the metals [58]. The activation entropy change is influenced by changes in the gradient of the density of states (DOS) curve at the Fermi level of the solid [26]. The calculations of the DOS of non-metal-modified nanoparticles are relatively difficult. As a consequence, it is interesting to relate the availability of electronic energy levels measured by experiment to the entropy change. Various orbitals for metal participate in covalent bond formation with surface-adsorbed species, offering an interpretation for the existence of weakly bound and strongly bound adsorbates [59]. XANES gives evidence of the *d*-band filling due to the presence of Pd–C and/or Pd–N in C-Pd/OMC and C,N-Pd/OMC interstitial catalysts. The filling of the *d* band of Pd, together with a pronounced change in the available electronic energy states, merely reflects the existence of a bound state for the absorbed intermediate that is energetically most favourable for the catalytic reaction [26]. In addition, the surface adsorption/desorption step is the rate-determining step in this strong adsorbate-involved reaction. The weakening of the adsorption of the strong adsorbate may increase the activity [60]. As a result, the *d* charge gain at Pd sites may indeed be responsible for the activation entropy change which is related to the adsorption configuration and strength, and therefore the optimal catalytic activity of Pd nanocatalysts. A linear relationship is established (Fig. 4b), which gives direct evidence of the surface adsorption-controlled direct bisarylation. The maximum *d*-charge gain which is obtained on the C and N co-doped Pd results in a pronounced change in the activation entropy, and apparently high direct bisarylation activity and stability in water.

In addition, when the N-free catalyst of C-Pd/OMC is compared to C,N-Pd/OMC, the exact role of N is clearer. Pd-N coordination can enhance the *d* charge at Pd site tuning the adsorption configuration and stabilize the surface Pd atoms free of leaching. As a result, both the activity and selectivity are increased.

### Heterogeneous vs. homogenous catalytic arylation mechanism

The mechanisms for homogenous Pd-catalysed arylation have been widely studied. The Pd^0/II^ catalysis may involve electrophilic attack of Pd on the arene, transmetallation and reductive elimination [10,16]; while the Pd^II/IV^ catalysis may involve the formation of a substrate-Pd^II^ complex through electrophilic palladation, and subsequent oxidative arylation (Supplementary Fig. 23) [61]. The single-site electron transfer process yields the monoarylated product.

**Figure 5.**
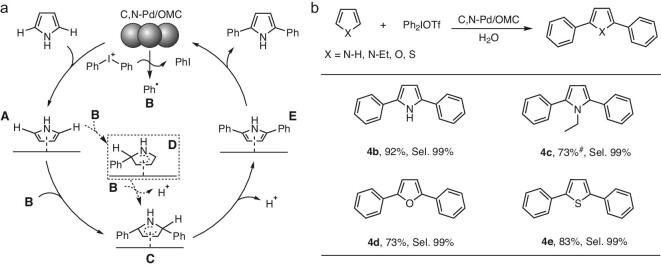
Possible mechanism and reaction scope. (a) Possible mechanism for the direct bisarylation over a heterogeneous C,N-Pd/OMC nanocatalyst with free pyrrole as an example. (b) Reaction possibilities for the direct bisarylation of five-membered heterocyclic compounds. The yield and the selectivity to bisarylated product are listed. Reaction conditions: 1 mol% catalyst; 0.2 mmol of substrate; 0.4 mmol Ph_2_IOTf; 2 mL H_2_O; 60°C; 8 h; atmospheric pressure; in air. *^#^*80°C, 12 h. Yields given are isolated yields.

In contrast, the surface reaction mechanism for the heterogeneous C,N-Pd/OMC interstitial catalysts is rather different. First, pyrrole adsorbs on the Pd clusters to form **A** (Fig. 5a). The flat-lying molecule lies over several Pd atoms and because it is almost symmetrical with C2 and C5 positions, is prone to electrophilic substitution at both positions for direct bisarylation. The modification of electronic properties here specified to be *d* charge gain at Pd sites, changes the activation entropy of the adsorbate, and therefore increases the catalytic activity. Simultaneously, the aryl radicals are formed by electron reduction of the diaryliodonium salts by Pd clusters [50]. A direct electrophilic addition between phenyl and adsorbed pyrrole at multi sites to form **C** is then proposed. Here a transient state of **D** is hypothesized, when the second aryl rapidly attacks the C5 site to give a **C**. Finally, after protonization, the bisarylation product is desorbed from the Pd surface to regenerate active Pd sites. The adsorption-induced C–H bond activation removes the limit on electron transfer on a single site of the homogenous catalyst.

According to the parallel-adsorption-induced direct bisarylation mechanism, the organic molecules, in which the π-bond of the ring coordinated with the Pd cluster, undergo direct bisarylation under the present conditions. *N*-ethylpyrrole, furan and thiophene were used as reactants (Fig. 5b). As expected, an ultra-high selectivity (99%) of bisarylated products is obtained with a high yield (73–92%).

## CONCLUSION

In summary, to overcome the restriction of electron transfer in the C–H functionalization over single-site homogenous catalysts, a direct bisarylation of heteroarenes has been realized for the first time over Pd nanocatalysts under green and mild conditions. The experimentally measured *d* charge on the transition metal site was used to correlate the activated entropy and catalytic activity. The maximum *d*-charge gain which was obtained on the C and N co-doped Pd resulted in a pronounced change in the activation entropy which is related to surface adsorption, and therefore an apparently high direct bisarylation activity and stability in water. The TOF value could reach 107 h^−1^ without detected by-products even for the direct bisarylation of free pyrrole under base-free, ligand-free and phase transfer agent-free conditions. The heterogeneous direct bisarylation demonstrates simultaneous multiple electron transfer and therefore an alternative synthesis path to the traditional homogenous process.

## METHODS

Methods are provided in the online Supplementary file.

## Supplementary Material

nwaa126_Supplemental_File
