# Supplementary material for: Unleash electron transfer in C–H functionalization by mesoporous carbon-supported palladium interstitial catalysts
Source: Natl Sci Rev. 2020 Jun 11;8(4):nwaa126. doi: 10.1093/nsr/nwaa126 (PMC8288372; doi:10.1093/nsr/nwaa126)
Supplement: nwaa126_Supplemental_File [file nwaa126_supplemental_file.docx]

**Unleash electron transfer in C-H functionalization by mesoporous carbon supported palladium interstitial catalysts**

**Xiaorui Zhao^1^, Yueqiang Cao^2^, Linlin Duan^1^, Ruoou Yang^3^ Zheng Jiang^3^, Chao Tian^1^, Shangjun Chen^1^, Xuezhi Duan^2^, De Chen^4^, Ying Wan^1,^***

^1^Key Laboratory of Resource Chemistry of Ministry of Education, Shanghai Key Laboratory of Rare Earth Functional Materials, and Department of Chemistry, Shanghai Normal University, Shanghai, P. R. China.

^2^State Key Laboratory of Chemical Engineering, East China University of Science and Technology, Shanghai, P. R. China.

^3^Shanghai Synchrotron Radiation Facility, Zhangjiang National Lab, Shanghai Advanced Research Institute, Chinese Academy of Sciences, Shanghai, China.

^4^Department of Chemical Engineering, Norwegian University of Science and Technology, Trondheim, Norway.

*Correspondence should be addressed to Y.W. (email: ywan@shnu.edu.cn)

**Contents**

**Supplementary Tables 3**

**Supplementary Figures 5**

**Supplementary Materials and Methods 29**

**^1^H NMR Data 33**

**Supplementary References 39**

**Supplementary Table 1**. The structural and textural properties of supported Pd catalysts.

| Sample | Pd loading*^a^* (wt%) | *τ^b^* (%) | *d*_Pd_*^b^* (nm) | *d*_Pd_*^c^* (nm) | *S*_BET_  (m^2^ g^-1^) | *V*_p_  (cm^3^ g^-1^) | *D*_p_  (nm) |
| --- | --- | --- | --- | --- | --- | --- | --- |
| C,N-Pd/OMC | 0.98 | 60 | 1.8 | 2.0 | 355 | 0.44 | 4.5 |
| C,N-Pd/OMC-R7 | 0.97 | 56 | 2.0 | 2.1 | 320 | 0.40 | 4.4 |
| 0.5% C,N-Pd/OMC | 0.51 | 60 | 1.8 | n.p.*^d^* | 343 | 0.43 | 4.5 |
| 1.5% C,N-Pd/OMC | 1.50 | 60 | 1.8 | n.p. | 330 | 0.42 | 4.5 |
| C-Pd/OMC | 1.00 | 49 | 2.2 | 2.4 | 325 | 0.50 | 7.8 |
| Pd/SBA-15 | 1.00 | 37 | 2.9 | 3.1 | 830 | 1.13 | 9.0 |
| Pd/C | 5.03 | 20 | 5.5 | 3.9 | 1333 | 0.82 | 1.4 |

*^a^* Measured by ICP-AES;

*^b^* Obtained from CO chemisorption, *τ* is the exposed surface atom dispersion, *d*_Pd_ is the average diameter of Pd nanoparticles;

*^c^* Estimated from the TEM images with the size histograms counted from at least 200 nanoparticles;

*^d^* Not provided.

**Supplementary Table 2.** *K*-edge *k*^2^ weighted EXAFS data fittings results of Pd samples.

| **Sample** | **Shell** | **CN*^a^*** | ***R*(Å)*^b^*** | **Δσ^2^*10^3^ (Å^2^)*^c^*** | ***r*-factor (%)*^d^*** |
| --- | --- | --- | --- | --- | --- |
| C,N-Pd/OMC | Pd–O/N | 1.1±0.2 | 1.98±0.02 | 3.6±2.5 | 0.1 |
|  | Pd–C | 1.1±0.3 | 2.17±0.02 | 3.4±3.1 |  |
|  | Pd–Pd | 5.3±1.1 | 2.77±0.01 | 9.3±1.6 |  |
| C,N-Pd/OMC-R7 | Pd–O/N | 1.6±0.2 | 2.00±0.02 | 6.6±0.9 | 0.3 |
|  | Pd–C | 1.0±0.1 | 2.19±0.03 | 4.6±2.1 |  |
|  | Pd–Pd | 4.9±1.3 | 2.77±0.02 | 8.9±2.5 |  |
| C-Pd/OMC | Pd–O | 0.3±0.1 | 2.00±0.02 | 6.5±0.7 | 0.7 |
|  | Pd–C | 1.4±0.2 | 2.19±0.02 | 5.0±0.6 |  |
|  | Pd–Pd | 6.4±0.9 | 2.78±0.01 | 7.8±1.2 |  |
| Pd foil | Pd–Pd | 12.0 | 2.75 | / | / |
| PdO | Pd–O | 4.0 | 2.02 | / | / |
|  | Pd–Pd | 4.0 | 3.03 | / |  |

*^a^*CN is the coordination number;

*^b^R* is the distance between absorber and backscattered atoms;

*^c^*∆σ^2^ is the Debye-Waller factor to account for both thermal and structural disorders;

*^d^r*-factor (%) indicates the goodness of the fit.

**Supplementary Fig. 1.** (**a**) N_2_ sorption isotherms and (**b**) Pore size distribution curves for C,N-Pd/OMC with different Pd loadings (0.51, 0.98 and 1.50 wt%). C,N-Pd/OMC-R7 is the C,N-Pd/OMC catalyst after seven catalytic runs.


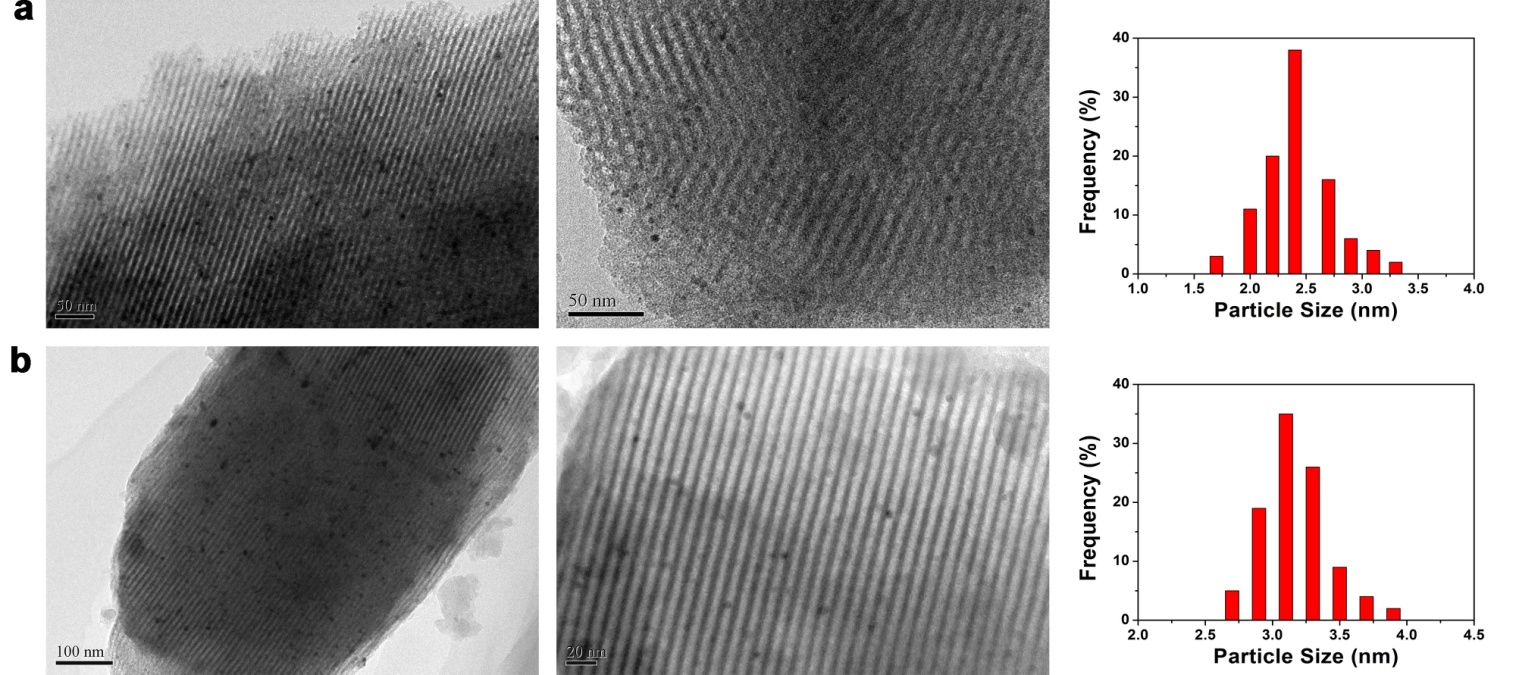


**Supplementary Fig. 2.** TEM images and particle size distribution histograms for (**a**) C-Pd/OMC and (**b**) Pd/SBA-15. The particle size distribution histograms of the catalyst was determined from at least 200 nanoparticles.

**
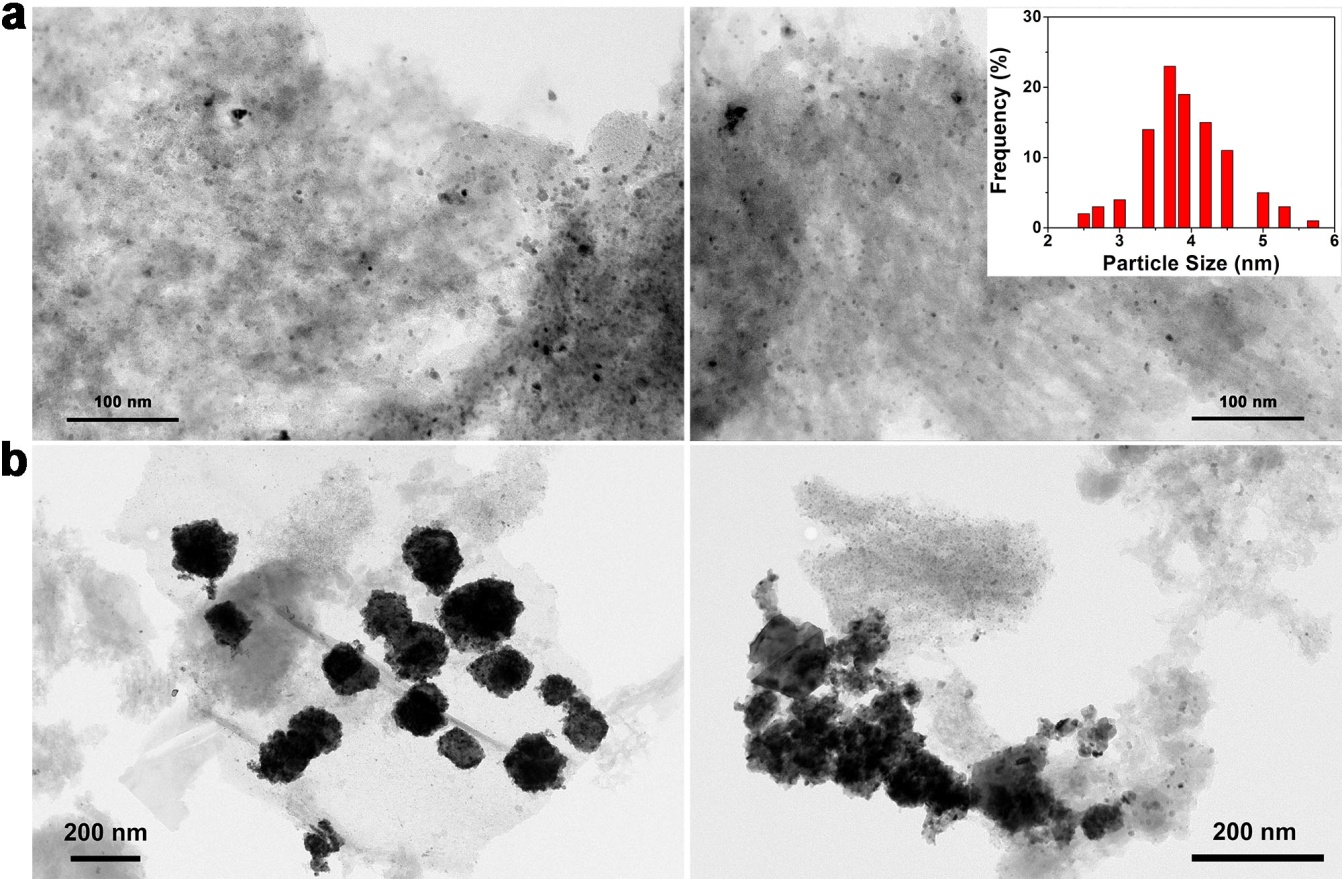
**

**Supplementary Fig. 3.** TEM images for (**a**) fresh and (**b**) used commercial Pd/C catalysts. The inset in (a) is the size distribution histogram counted from at least 200 nanoparticles.

**Supplementary Fig. 4.** (**a**) N_2_ sorption isotherms and (**b**) pore size distribution curves for various solid Pd nanocatalysts: C-Pd/OMC, Pd/SBA-15 and commercial Pd/C.

**Supplementary Fig. 5.** Temperature programmed reduction-mass spectrometry (TPR-MS) for as-made C,N-Pd/OMC-as (the support after impregnation with Pd solution but without H_2_ reduction). The sample (ca. 50 mg) was purged at 100 ºC for 1 h and then cooled to 50 ºC in flowing dry Ar (30 ml min^−1^, >99.999%). The sample was then heated to 300 ºC at a rate of 5 ºC min^−1^ in flow of 10 v% H_2_ in Ar, 30 ml min^−1^ and maintained at 300 ºC for 180 mins. The signals of mass-to-charge (m/z) ratios of 16, 18, 28, 30, 44 and 46 were recorded.

**Supplementary Fig. 6.** XPS spectra of the 1*s* level of N for as-made N-containing OMC carrier (N-OMC-as), OMC-as carrier heated at 275 ºC (N-OMC), as-made C,N-Pd/OMC (C,N-Pd/OMC-as) and C,N-Pd/OMC.

**Supplementary Fig. 7.** Effect of Pd loading on the reaction rate of direct bisarylation of *N*-methylpyrrole using the C,N-Pd/OMC catalyst (Madon-Boudart test [1]). *f*_m_ is the concentration of surface active metal material. Reaction conditions: 21 mg of catalyst with the Pd concentration range of 0.51 – 1.50 wt% ; 0.2 mmol of *N*-methylpyrrole; 0.4 mmol of Ph_2_IOTf, 2 ml of H_2_O; 60 ºC; 800 rpm; atmospheric pressure; in air.

**Supplementary Fig. 8.** Bisarylation pathways of *N*-methylpyrrole catalyzed by heterogeneous Pd*_n_*^0^ cluster or homogenous Pd^2+^ catalysts.

**Supplementary Fig. 9.** Effect of *N*-methylpyrrole concentrations on the reaction rate for the direct bisarylation of *N*-methylpyrrole over the C,N-Pd/OMC catalyst. Reaction conditions: 0.002 mmol Pd catalyst; 2 ml of water; 60 ºC; 800 rpm; atmospheric pressure; in air.

**Supplementary Fig. 10.** Arrhenius plots for the direct bisarylation of *N*-methylpyrrole over the solid C,N-Pd/OMC, C-Pd/OMC, Pd/SBA-15 and Pd/C catalysts.

**Supplementary Fig. 11.** The yield plot for *N*-methyl-2,5-diphenylpyrrole over the C,N-Pd/OMC catalyst in the hot filtration experiment (red line). The C,N-Pd/OMC catalyst was removed by hot filtration after 30-min reaction at 60 ºC. Fresh substrate and Ph_2_IOTf were then added to the filtrate, while the reaction temperature was kept at 60 ºC. For comparison, the yield plot for the *N*-methyl-2,5-diphenylpyrrole over the C,N-Pd/OMC catalyst along with the reaction time is also provided.

**Supplementary Fig. 12.** Compilation of the (**a**) initial reaction rate and selectivity to the diarylated product of *N*-methyl-2,5-diphenylpyrrole and (**b**) yield of *N*-methyl-2,5-diphenylpyrrole for the direct bisarylation of *N*-methylpyrrole in successive runs over the recovered C,N-Pd/OMC catalyst. Reaction conditions: 1 mol% Pd catalyst; 0.2 mmol of *N*-methylpyrrole; 0.4 mmol of Ph_2_IOTf; 2 ml of water; 8 h; 60 ºC; 800 rpm; atmospheric pressure; in air.

**Supplementary Fig. 13.** XPS spectra of the 3*d* level of Pd for used solid catalysts: C,N-Pd/OMC-R7 and C-Pd/OMC-R.

**Supplementary Fig. 14.** Yields for *N*-methyl-2-phenyllpyrrole and *N*-methyl-2,5-diphenylpyrrole with reaction time in the Pd-catalyzed arylation of *N*-methylpyrrole: (**a**) Pd(OAc)_2_; (**b**) Pd/C; (**c**) Pd/SBA-15; and (**d**) C-Pd/OMC. Reaction conditions: 1 mol% Pd catalyst; 0.2 mmol of *N*-methylpyrrole; 0.4 mmol of Ph_2_IOTf; 2 ml of water; 60 ºC; 800 rpm; atmospheric pressure; in air.


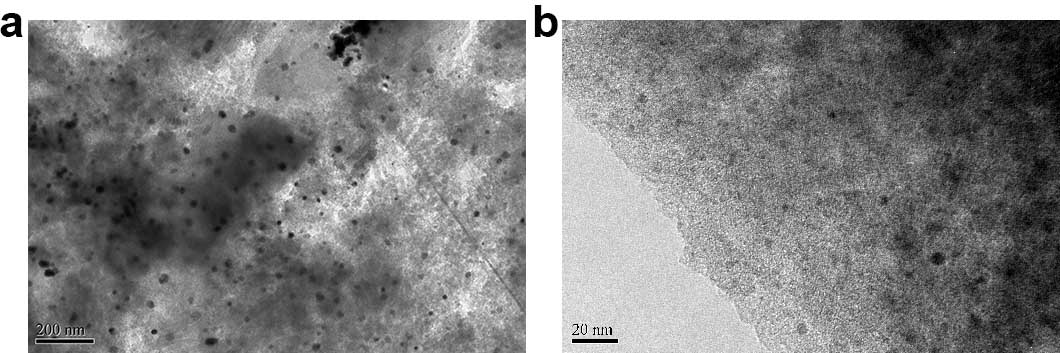


**Supplementary Fig. 15.** TEM images with (**a**) low and (**b**) high magnifications of the aggregated Pd species generated during the Pd(OAc)_2_-catalyzed reaction and captured by activated carbon. 10 mg of activated carbon was added to the batch after 1-h reaction to capture the aggregated Pd species. After filtration, washing and drying, the activated carbon was characterized. Reaction conditions: 1 mol% Pd catalyst; 0.2 mmol of *N*-methylpyrrole; 0.4 mmol of Ph_2_IOTf; 2 ml of water; 60 ºC; 800 rpm; atmospheric pressure; in air.

**Supplementary Fig. 16.** Yields for *N*-methyl-2-phenylpyrrole and *N*-methyl-2,5-diphenylpyrrole with reaction time in the Pd(OAc)_2_-catalyzed arylation of *N*-methylpyrrole with a substrate:Pd ratio of 1000:1. Reaction conditions: 0.1 mol% of Pd; 0.2 mmol of *N*-methylpyrrole; 0.4 mmol of Ph_2_IOTf; 2 ml of water; 60 ºC; 800 rpm; atmospheric pressure; in air.


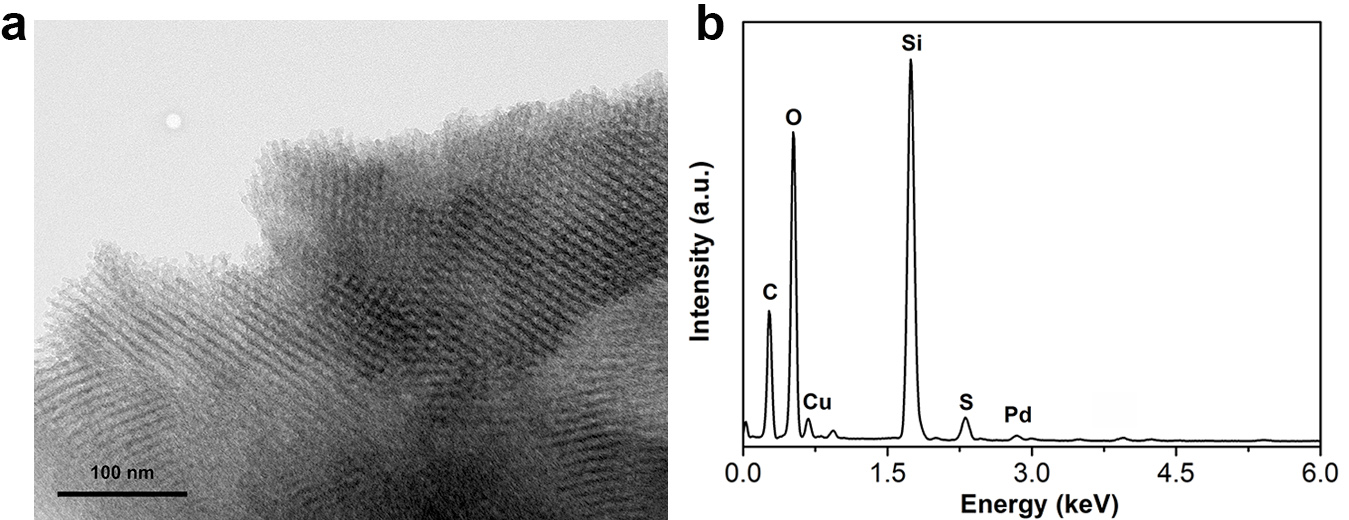


**Supplementary Fig. 17.** (**a**) TEM image and (**b**) EDS pattern for Pd/SH-SBA-15 produced during the solid trapping test using SH-SBA-15 for the Pd/C catalyzed reaction. The solid trapping agent SH-SBA-15 was added to the reaction mixture containing Pd/C (1 mol% of Pd), 0.2 mmol of *N*-methylpyrrole, 0.4 mmol of Ph_2_IOTf and 2 ml of water. After stirring for 8 h at 60 ºC, the solid was separated, washed, dried, and characterized. Domains with ordered mesopore arrays were thoroughly investigated and typical results are shown.

**Supplementary Fig. 18.** Radical trapping experiments. Reaction conditions: 1 mol% Pd; 0.2 mmol of *N*-methylpyrrole; 0.4 mmol of radical scavenger (2,2,6,6-Tetramethyl-1-piperidinyloxy (TEMPO), 1,4-Benzoquinone (BQ), 2,6-Di-tert-butyl-4-methylphenol (BHT)); 0.4 mmol of Ph_2_IOTf; 2 ml of water; 60 ºC; 8 h; 800 rpm; atmospheric pressure; in air.

**Supplementary Fig. 19.** Deuteration experiment of *N*-methylpyrrole in the absence of a coupling partner. Reaction conditions: 1 mol% Pd; 0.2 mmol of *N*-methylpyrrole; 1 ml of deuterium reagent (D_2_O or CD_3_OD); 1 ml of water; 8 h; 60 ºC; 800 rpm; atmospheric pressure; in air. The yield was determined by ^1^H nuclear magnetic resonance spectra (NMR).

**Supplementary Fig. 20.** Kinetic isotope effect (KIE) experiments of the parallel reactions. Reaction conditions: 1 mol% of Pd; 0.2 mmol of pyrrole or pyrrole-*d*5 (95%-*d*); 0.4 mmol of Ph_2_IOTf; 2 ml of water; 60 ºC; 800 rpm; atmospheric pressure; in air. The yields were determined by ^1^H NMR.

**Supplementary Fig. 21.** KIE experiment of the competitive reaction. Reaction conditions: 1 mol% of Pd; 0.1 mmol of pyrrole; 0.1 mmol of pyrrole-*d*5 (95%-*d*); 0.4 mmol of Ph_2_IOTf; 2 ml of water; 60 ºC; 800 rpm; atmospheric pressure; in air. The yields were determined by ^1^H NMR.

**Supplementary Fig. 22.** Relationship between the TOF value and the apparent activation entropy for the direct bisarylation of *N*-methylpyrrole over Pd catalysts supported on various carriers.

**Supplementary Fig. 23.** Possible homogenous catalysis mechanisms of the arylation of *N*-methylpyrrole: (**a**) Pd^0/II^-involved and (**b**) Pd^II/IV^-involved catalysis [2,3].

**Supplementary Fig. 24.** Synthesis and characterization of mercapto-functionalized ordered mesoporous silica SH-SBA-15. (**a**) FT-IR spectra for pristine and mercapto-functionalized SH-SBA-15. Compared to that of pristine SBA-15, the FTIR spectrum of SH-SBA-15 contained several absorbances in the 3000 – 2800 cm^-1^ range, which were assigned to the C–H vibrations in the mercaptopropyl groups. This phenomenon provides further evidence of the modification of the organic functional groups on mesoporous silica SBA-15. However, the mercapto group is IR invisible under the current conditions, which may be due to the weak dipoles of the S–H groups that hinder detection of their modes by vibrational spectroscopy. (**b**) TG curves for pristine and mercapto-functionalized SH-SBA-15. The TG curve for pristine SBA-15 showed a weight loss below 100 ºC, corresponding to the physisorption of water. In comparison, the mercapto-functionalized SBA-15 showed a second distinct weight loss between 250 and 500 ºC, which may be due to the loss of grafted mercaptopropyl groups on the mesoporous silica. The S content was estimated to be approximately 2.5 mmol S g^−1^ solid, which is consistent with the value obtained by elemental analysis. (**c**) XPS spectrum for SH-SBA-15 in the S 2*p* region, showing one strong peak corresponding to the –SH group. (**d**) N_2_ sorption isotherms for pristine and mercapto-functionalized SH-SBA-15. Typical type IV curves are observed for both samples, suggesting uniform mesopores. After modification of the mercaptopropyl groups, the BET surface area and pore volume decreased from 706 to 510 m^2^ g^−1^, and from 1.04 to 0.78 cm g^−1^, respectively, and the pore size remained at 8.9 nm. The results are similar to those of mesoporous silica modified with organic moieties and reflect grafting of the moieties inside the pores [4,5].

**Supplementary Materials and Methods.**

**Materials**. Octadecyldimethyl[3-(trimethoxysilyl)propyl]ammonium chloride (60% in methanol), Na_2_PdCl_4_ (98%), PdCl_2_(98%), Pd(OAc)_2_ (99%), and 5% Pd/C (wetted with ca. 55% water) were purchased from Meryer Chemical Technology Co.; triblock copolymer Pluronic P123 (poly(propylene oxide)-*bl*oc*k*-poly(ethylene oxide)-*bl*oc*k*-poly(propylene oxide), PEO_20_PPO_70_PEO_20_, 5,800) and PluronicF127 (PEO_106_PPO_70_PEO_106_, 12,600) were purchased from Aldrich Chemical Co; and mercapto-functionalized silica (SH-silica, S loading：1.6 mmol g^−1^), and diphenyliodonium triflate (98%) were purchased from J&K Chemical Co. Other chemicals were obtained from the Shanghai Chemical Company. All chemicals were used as received without any further purification. Water used in all experiments was deionized.

**Synthesis of SBA-15**. Pristine mesoporous silica SBA-15 was synthesized by a hydrothermal method [6]. In a typical synthesis, 1.0 g of P123 block copolymer was dissolved with stirring in a solution of 7.5 g of water and 30.0 g of HCl (2 M), and then 2.08 g of TEOS was added with stirring at 40 ºC. After being stirred for 20 h at 40 ºC, the gel solution was transferred into a Teflon bottle. The hydrothermal temperature and time were 100 ºC and 24 h, respectively. The SBA-15 carrier was obtained after removing the surfactant at 550 ºC under air.

**Synthesis of SH-SBA-15**. SH-SBA-15 was synthesized by grafting mercaptopropyl groups on pre-prepared mesoporous silica SBA-15 [5]. A toluene (120 ml) suspension of SBA-15 (4.1 g) was then mixed with 10.0 g of 3-thiolpropyltrimethoxysilane at 110 ºC with reflux for 48 h; 1.8 ml of water was added to promote cross-linking, and the mixture was heated at reflux for an additional 24 h. The solids were then filtered and washed with copious amounts of toluene, hexanes, and methanol to remove unreacted silanes. The recovered solids were Soxhlet extracted with dichloromethane at reflux temperature for 24 h. The resulting white solids were collected, dried at room temperature overnight and subsequently at 150 ºC for 3 h under vacuum, and stored in a vacuum dryer.

**X-ray absorption data analysis.** The X-ray absorption fine structure (XAFS) spectra for Pd *K*-edge data were obtained at the BL14W1 of the Shanghai Synchrotron Radiation Facility (SSRF). The samples were pressed into pellets and measured in the fluorescence mode. A double-crystal Si(311) monochromator was used for energy selection at the Pd *K*-edge (24,350 eV). The acquired XAFS data were processed according to standard procedures using the ATHENA module implemented in the IFEFFIT software packages [7]. The *k*^2^-weighted EXAFS spectra were obtained by subtracting the post-edge background from the overall absorption and then normalizing with respect to the edge-jump step. Subsequently, *k*^2^-weighted *χ*(*k*) data in the *k* space ranging from 3.0 to 12.0 Å^-1^ were Fourier-transformed to real (*R*) space using a Hanning window (d*k*= 1.0 Å^−1^) to separate the EXAFS contributions from the different coordination shells. The obtained amplitude reduction factor S_0_^2^ of Pd foil was 0.77 and it was fixed in the subsequent fitting of the Pd foil *K*-edge data for the catalyst. To obtain the quantitative structural parameters around the central atoms, least-squares curve parameter fitting was performed using the ARTEMIS module of the IFEFFIT software packages. The following EXAFS equation was used:

$\begin{matrix} \chi(k)=\sum N_{\text{j}}F_{j}(k)\exp(-2\sigma_{j}^{2}k_{j}^{2})\sin(2krj+\varphi_{j}(k))/kr_{j}^{2} \\ k_{j}=(k^{2}-2m\Delta E_{0}/h^{2})^{1/2} \end{matrix}$ (1)

where *N_j_*, *r_j_, σ_j_*, and Δ*E_0j_* represent the coordination number, the bond distance, the Debye-Waller factor, and the energy shift between reference and sample, respectively [8].

The XAFS spectra for Pd *L*_3_-edge data were conducted at the 4B7A station in the Beijing Synchrotron Radiation Facility (BSRF). A double-crystal Si(111) monochromator was used for energy selection at the Pd *L*_3_-edge (3173 eV). The EXAFS spectra were obtained by subtracting the post-edge background from the overall absorption and then normalizing with respect to the edge-jump step. The difference in the number of 4*d* holes (*d*-charge gain) between the samples and Pd metal was evaluated from the Pd *L*-edges XANES using the following equation: Δ*n_d_* = [(*A_sample_* – *A_Pd_*)Pd*L_3_* + (*A_sample_* – *A_Pd_*)Pd*L*_2_]/10.45, where *A* is the peak area of the white lines at the *L*_3_- or *L*_2_-edges, and 10.45 is the absorption cross-section per hole in 4*d* band of each Pd atom [9-11]. In order to eliminate the size effect, Pd/SBA-15 was used as the reference of the Pd metal. The ratio of *L*_3_ peak area to *L*_2_ peak area was determined to be about 2.5 according to the literature for simplification [12].

**DFT calculations.** All the density functional theory (DFT) calculations in this work were performed by using the Vienna Ab-initio Simulation Package (VASP) [13,14]. The generalized gradient approximation (GGA) with the Perdew-Burke-Ernzerhof (PBE) functional was used to describe the electronic exchange and correlation eﬀects [15]. A plane wave basis sets with a cutoff energy of 400 eV was used to expand the solution of the Kohn-Sham equations. The Brillouin zone was sampled by a 3×3×1 Monkhorst-Pack k-point grid [16]. The geometry optimization was converged to the force on each atom less than 0.03 eV/Å and the total energy smaller than 10^−6^eV. The Pd(111) surface was modeled using a supercell of (2×2) with five atomic layers, and the Pd(211) surface was modeled using a super cell of (2×2) with four atomic layers. The bottom two layers were fixed at the bulk position during the geometry optimization. A vacuum layer of 15 Å was used to avoid the interaction between the neighboring slabs. The surface and subsurface carbon atom modified surfaces were modeled by optimizing the position of the carbon atoms on the surface and subsurface, respectively. The surface C preferred to locate at the trigonal hollow *hcp* site of Pd(111), while the subsurface C preferred to locate at the octahedral subsurface sites of Pd surfaces. These results were in good agreement with previous studies [17,18]. The adsorption energies of pyrrole on the clean surfaces, surface and subsurface carbon atoms modified surfaces (*E*_ads_) were calculated as follows: *E*_ads_ = *E*_pyrrole/surface_ – *E*_pyrrole_ –*E*_surface_, where *E*_pyrrole/surface_ and *E*_surface_ are the total energies of the surface with and without the pyrrole adsorbate, and *E*_pyrrole_ is the energy of a free pyrrole molecule.

**Catalysis tests.** The scaled-up reactions were carried out in a 50 ml round-bottled flask under 60 ºC by using water as the solvent. 180 μl (2.0 mmol) of *N-*methylpyrrole, 1.72 g (4.0 mmol) of Ph_2_IOTf were added to 20 ml of water and placed in the reactor. To this, 0.21 g (containing 0.02 mmol metallic Pd) of supported palladium catalyst was added. The mixture was heated to 60 ºC, with a stirring speed of 800 rpm.

The monoarylated product (*N*-methyl-2-phenylpyrrole) was also used as a reactant for parallel and competitive experiments with *N*-methylpyrrole. In parallel experiments, 21 mg of C,N-Pd/OMC catalyst, 0.2 mmol of *N*-methylpyrrole or *N*-methyl-2-phenylpyrrole, 0.4 mmol of Ph_2_IOTf and 2 ml of water were added in a 25 ml round-bottled flask, and the mixture was heated to 60 ºC. In the competitive experiment, 21 mg of C,N-Pd/OMC catalyst, 0.1 mmol of *N*-methylpyrrole, 0.1 mmol of *N*-methyl-2-phenylpyrrole, 0.4 mmol of Ph_2_IOTf and 2 ml of water were mixed.

For radical trapping tests, radical scavenger (2,2,6,6-tetramethyl-1-piperidinyloxy (TEMPO), 1,4-benzoquinone (BQ), or 2,6-di-tert-butyl-4-methylphenol (BHT)) was added to the reaction (scavenger:*N*-methylpyrrole = 2 in molar ratio) containing the substrate and catalyst at 60 ºC, with a stirring speed of 800 rpm for 8 h.

**Kinetics calculations**. The turn over frequency (TOF) for the C,N-Pd/OMC catalyst was calculated on the basis of the estimated number of exposed palladium atoms, at a conversion below 20%.

$\text{TOF}_{\text{Pd}}=\frac{n_{\text{Sub}}X}{n_{\text{Pd}}t\tau}$ (2)

where *n*_Sub_ is the molar amount of the substrate, *X* is the conversion, *n*_Pd_ is the molar amount of Pd, *t* is the reaction time, and *τ* is the exposed surface atom dispersion.

In the cases of Pd/C, and Pd/SBA-15, the contribution of conversion from the leached Pd was first subtracted by assuming that the monoarylated product was produced by the presence of homogenous leached Pd and the bisarylated product were produced on their derived Pd*_n_*^0^ clusters. The Pd^2+^ concentration was determined on the basis of the reaction rate over dilute Pd(OAc)_2_. In the initial period, the reaction rate (*r*_0_ = 192 mmol mmol_Pd_^−1^ h^−1^) and the selectivity (> 99%) to *N*-methyl-2-phenylpyrrole were extremely high. As a result, all Pd^2+^ in dilute Pd(OAc)_2_ possibly exclusively contributed to the initial reaction rate for monoarylation. Then, the Pd^2+^ concentration which contributed to the monoarylation in Pd/C- and Pd/SBA-15-catalyzed system could be estimated. The initial reaction rate for the direct bisarylation by the generated Pd*_n_*^0^ from leached Pd^2+^ was calculated by the yield to the bisarylated product divided by the difference between total Pd and Pd^2+^ concentration and the reaction time. The reaction rate for the generated Pd*_n_*^0^ from in Pd/C- and Pd/SBA-15-catalyzed system was estimated to be 15.3 and 13.5 mmol mmol_Pd_^−1^ h^−1^, respectively. These values were very close to the second step in the low-concentration-Pd(OAc)_2_-catalyzed reaction, where the aggregated Pd*_n_*^0^ clusters from Pd^2+^ were formed and resulted in the yield of bisarlated product of *N*-methyl-2,5-diphenylpyrrole (13 mmol mmol_Pd_^−1^ h^−1^), implying the reasonability of the assumption. The Pd*_n_*^0^ clusters were small which have been well documented, and the surface exposure was estimated to be close to 100%. Therefore, the TOF value was equal to the initial reaction rate.

For the C-Pd/OMC catalyst, the leached Pd was limited, since the solid trapping agent SH-SBA-15 could only quench the yield of monoarylated product (7%) and showed a negligible effect on the production of bisarylated product (58%). Therefore, the initial reaction rate for the direct bisarylation by the carbon-modified Pd nanoparticles was calculated by the yield to the bisarylated product divided by the difference between total Pd and Pd^2+^ concentration, and the reaction time. The Pd^2+^ concentration which contributed to the monoarylation could be estimated by *r*_0,Pd2+_ of 192 mmol mmol_Pd_^−1^ h^−1^, and the obtained concentration was 8.9% to the total Pd in molar ratio. This value was very close to that obtained from the thorough analysis by XPS. The surface exposure was adapted to the value for fresh C-Pd/OMC. The TOF value was estimated to be 58.3 h^−1^.

Apparent activation energies (*E*_a_) were calculated according to the Arrhenius equation:

$\ln k=\ln A-\frac{E_{a}}{RT}$ (3)


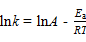


where *k* is the reaction rate constant, *A* is the apparent pre-exponential factor, *R* is the universal gas constant and *T* is the reaction temperature. Taking into account the approximate zero-order reaction kinetics for bisarylation of *N*-methylpyrrole, *k* is simplified to be close to initial reaction rate *r*_0_.

The apparent enthalpy of activation (Δ*S*^0*^) was determined as follows.

The TOF value was expressed in the Eyring form [19,20]:

$\text{TOF}_{\text{Pd}}=\frac{k_{\text{B}}T}{h}\exp(\frac{\Delta S^{0*}}{R})\text{exp}(-\frac{\Delta H^{0*}}{RT})$ (4)

where *k*_B_, *h*, Δ*S*^0*^, and Δ*H*^0*^ is the Boltzmann constant, Planck constant, entropy of activation, and enthalpy of activation, respectively.

The apparent activation energy *E*_a_ was related to Δ*H*^0*^ by the Temkin equation:

$E_{a}=\Delta H^{0*}+\sum n_{i}\Delta H_{i}$ (5)

where Δ*H_i_* and *n_i_* is the adsorption enthalpy and the reaction order of reactant *i*, respectively. Taking into account the approximate zero-order reaction kinetics for bisarylation of *N*-methylpyrrole, *E*_a_ was simplified to be close to Δ*H*^0*^.

The entropy change in the activation step of the chemical reaction was closely related to the thermodynamics of the rate constant, which results in the following equation:

$\Delta S^{0*}=R\ln(\frac{Ah}{k_{\text{B}}T\tau})$ (6)

**^1^H NMR Data.**

**1-Methyl-5-phenyl-1*H*-pyrrole (3a).** Palladium acetate (1 mol% of Pd), *N*-methyl-1*H*-pyrrole (**1a**) (1.0 mmol), diphenyliodonium triflate (**2a**) (2.0 mmol), and deionized water (10.0 ml) were added in a 25 ml vial. The solution was heated with stirring to 60 ºC for 8 hours. **3a** was obtained as a white solid (48 mg, 31% yield) after purification by column chromatography (hexanes/ethyl acetate 98/2). *R_f_* = 0.28 (hexanes/ethyl acetate 95/5). **^1^H NMR (400 MHz, CDCl_3_, ppm)**: δ 7.40 – 7.25 (m, 5H), 6.72 – 6.71 (dd, *J* = 4.6, 2.8 Hz, 1H), 6.24 – 6.23 (m, 1H), 6.22 – 6.20 (m, 1H), 3.67 (s, 3H). The ^1^H NMR data were in accordance with literature [21].


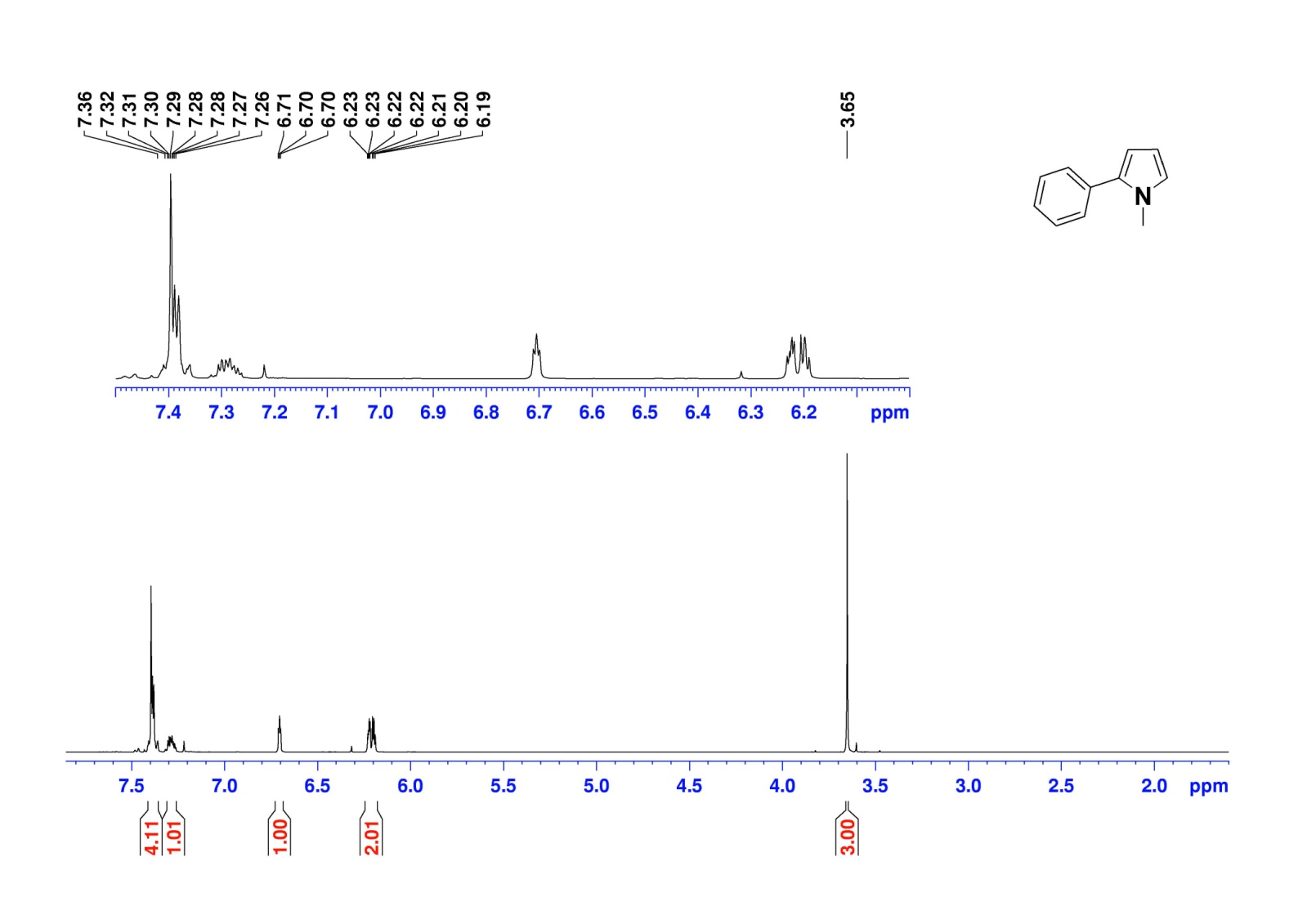

**1-Methyl-2,5-diphenyl-1*H*-pyrrole (4a).** Palladium acetate (1 mol% of Pd), *N*-methyl-1*H*-pyrrole (**1a**) (1.0 mmol), diphenyliodonium triflate (**2a**) (2.0 mmol), and deionized water (10.0 ml) were added in a 25 ml vial. The solution was heated with stirring to 60 ºC for 36 hours. **4a** was obtained as a light yellow solid (149 mg, 64% yield) after purification by column chromatography (hexanes/ethyl acetate 98/2). **^1^H NMR (400 MHz, CDCl_3_, ppm)**: δ 7.49 – 7.47 (d, 4 H), 7.44 – 7.40 (t, *J* = 7.5, 3.4 Hz, 4 H), 7.32 – 7.29 (t, *J* = 7.3 Hz, 2 H), 6.32 (s, 2 H), 3.61 (s, 3 H). The ^1^H NMR data were accordance with literature [22].


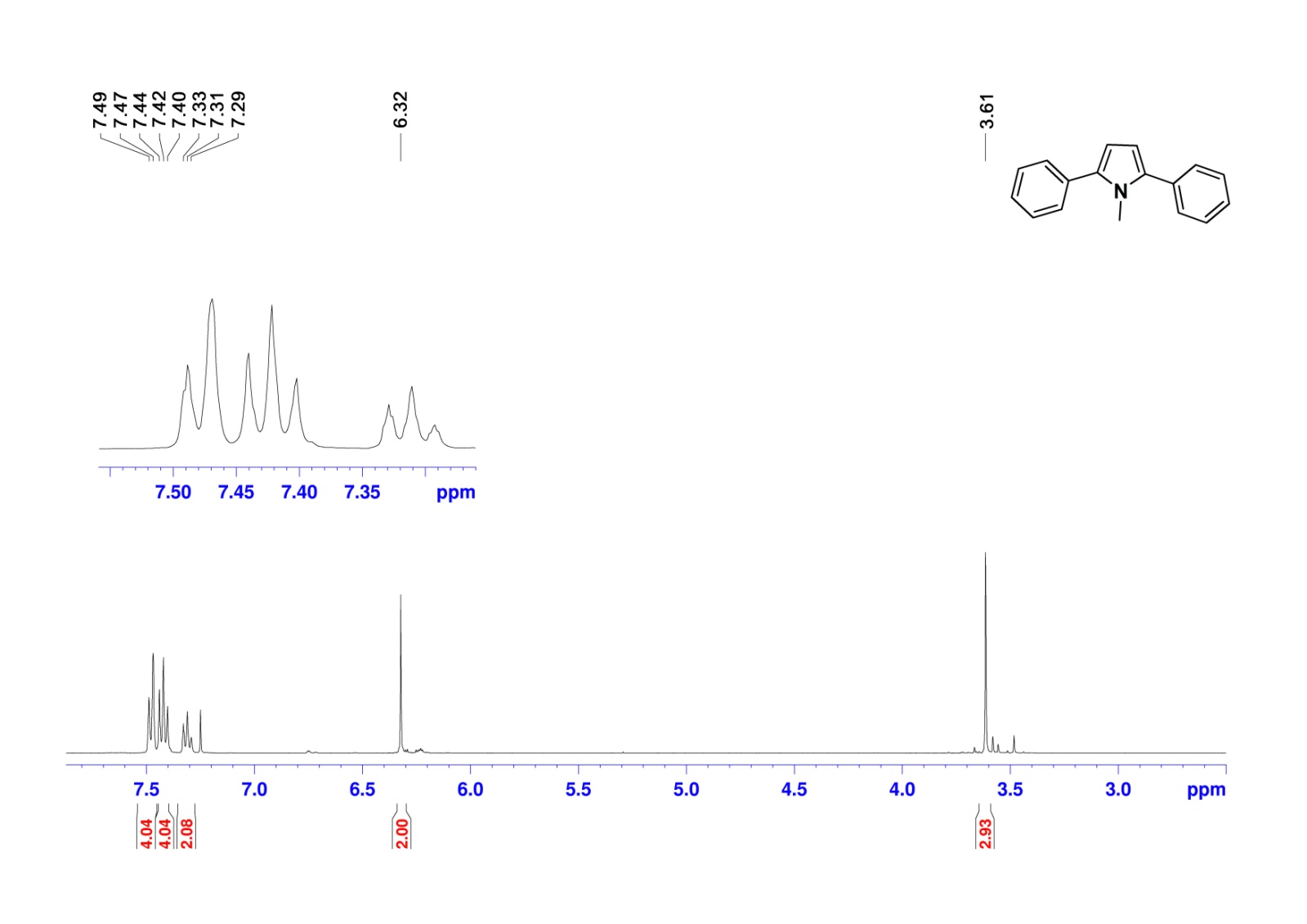

**2,5-Diphenyl-1*H*-pyrrole (4b).** C,N-Pd/OMC (1 mol% of Pd), *N*-methyl-1*H*-pyrrole (**1b**) (0.2 mmol), diphenyliodonium triflate (**2a**) (0.4 mmol), and deionized water (2.0 ml) were added in a 10 ml vial. The solution was heated with stirring to 60 ºC for 8 hours. **4b** was obtained as a white solid (40 mg, 92% yield) after purification by column chromatography (hexanes/ethyl acetate 90/10). **^1^H NMR (400 MHz, CDCl_3_, ppm)**: δ 8.61 (br s, 1H), 7.45 (m, 4H), 7.41 – 7.37 (m, 4H), 7.26 – 7.22 (m, 2H), 6.58 (d, *J* = 2.4 Hz, 2H). The ^1^H NMR data were in accordance with literature [23].

**^
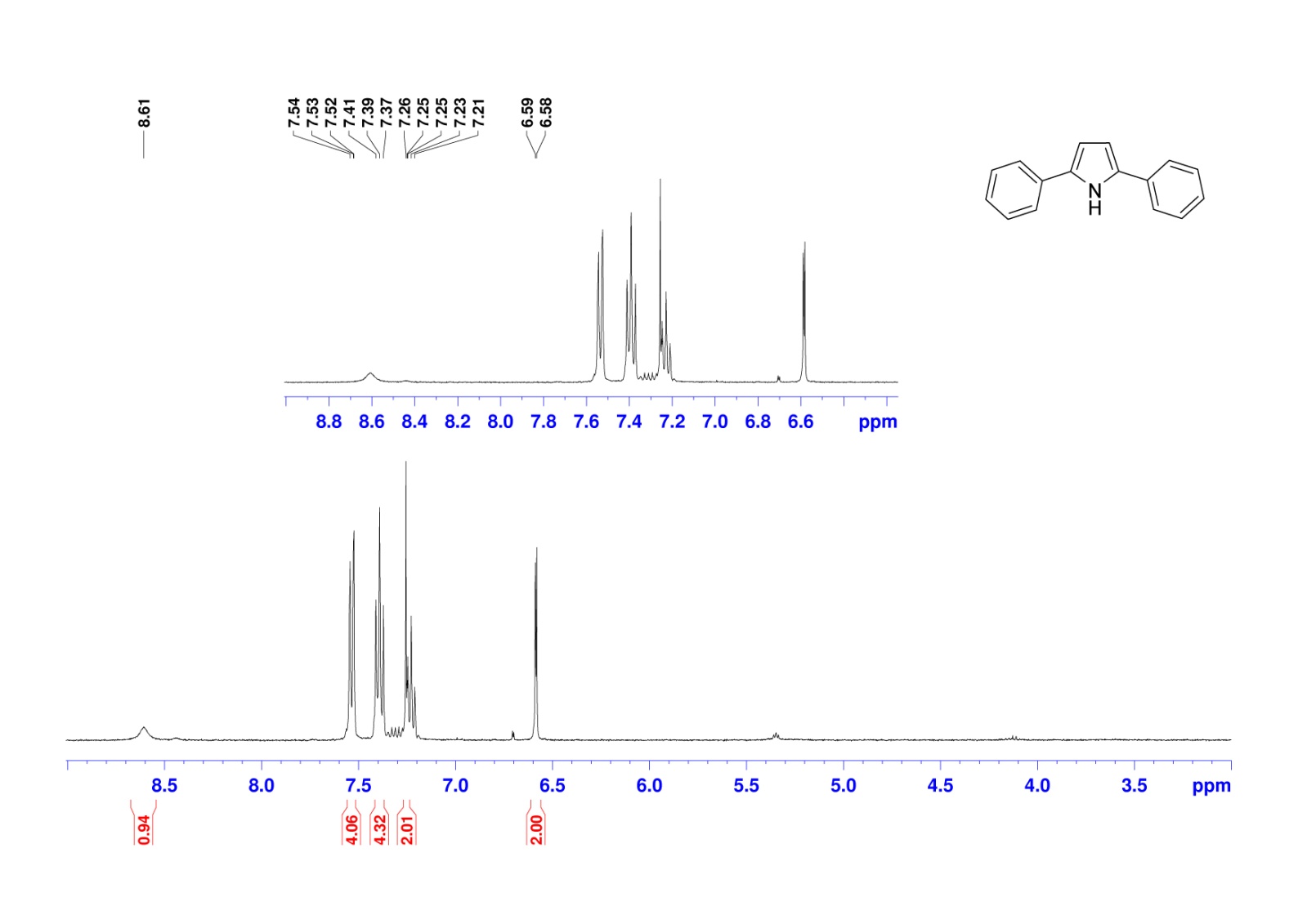
^**

**1-Ethyl-2,5-diphenyl-1*H*-pyrrole (4c).** C,N-Pd/OMC (1 mol% of Pd), 1-ethyl-1*H*-pyrrole (**1c**) (0.2 mmol), diphenyliodonium triflate (**2a**) (0.4 mmol), and deionized water (2.0 ml) were added in a 10 ml vial. The solution was heated with stirring to 80 ºC for 12 hours. **4c** was obtained as a pale red solid (36 mg, 73% yield) after purification by column chromatography (hexanes/ethyl acetate 95/5). **^1^H NMR (400 MHz, CDCl_3_, ppm)**: δ 7.51 – 7.49 (m, 4H), 7.47 – 7.43 (m, 4H), 7.36–7.34 (m, 2H), 6.29 (s, 2H), 4.16 – 4.11 (m, 2H), 0.91 – 0.88 (m, 3H). The ^1^H NMR data were in accordance with literature [22].


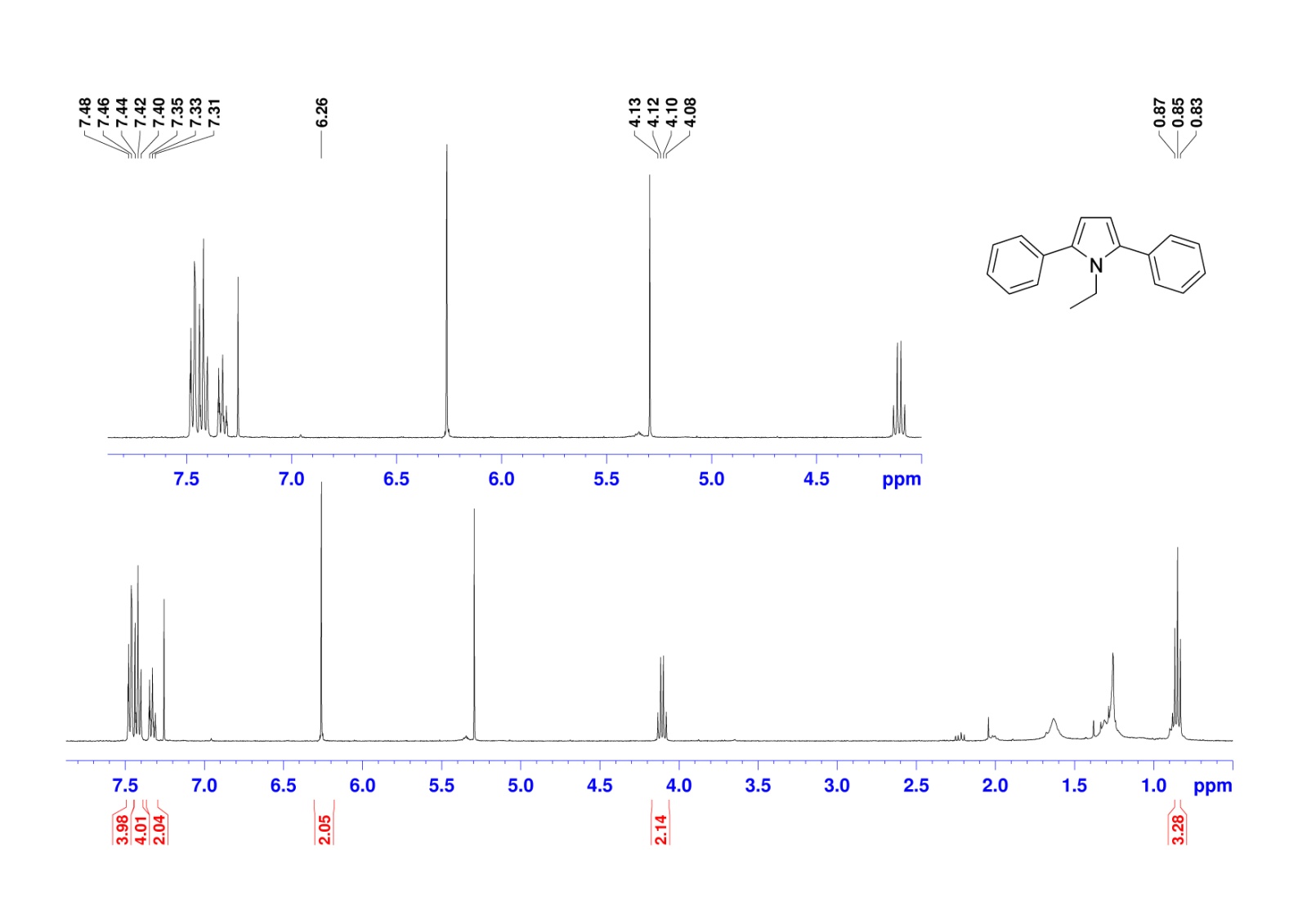

**2,5-Diphenylfuran (4d).** C,N-Pd/OMC (1 mol% of Pd), furan (**1d**) (0.2 mmol), diphenyliodonium triflate (**2a**) (0.4 mmol), and deionized water (2.0 ml) were added in a 10 ml vial. The solution was heated with stirring to 60 ºC for 8 hours. **4d** was obtained as a white solid (32 mg, 73% yield) after purification by column chromatography (hexanes/ethyl acetate 90/10). **^1^H NMR (400 MHz, CDCl_3_, ppm)**: δ = 7.74 (dd, *J* = 8.0, 1.2 Hz, 4H), 7.43 – 7.39 (t, 4H), 7.29 – 7.25 (t, 2H), 6.75 (s, 2H). The ^1^H NMR data were in accordance with literature [24].


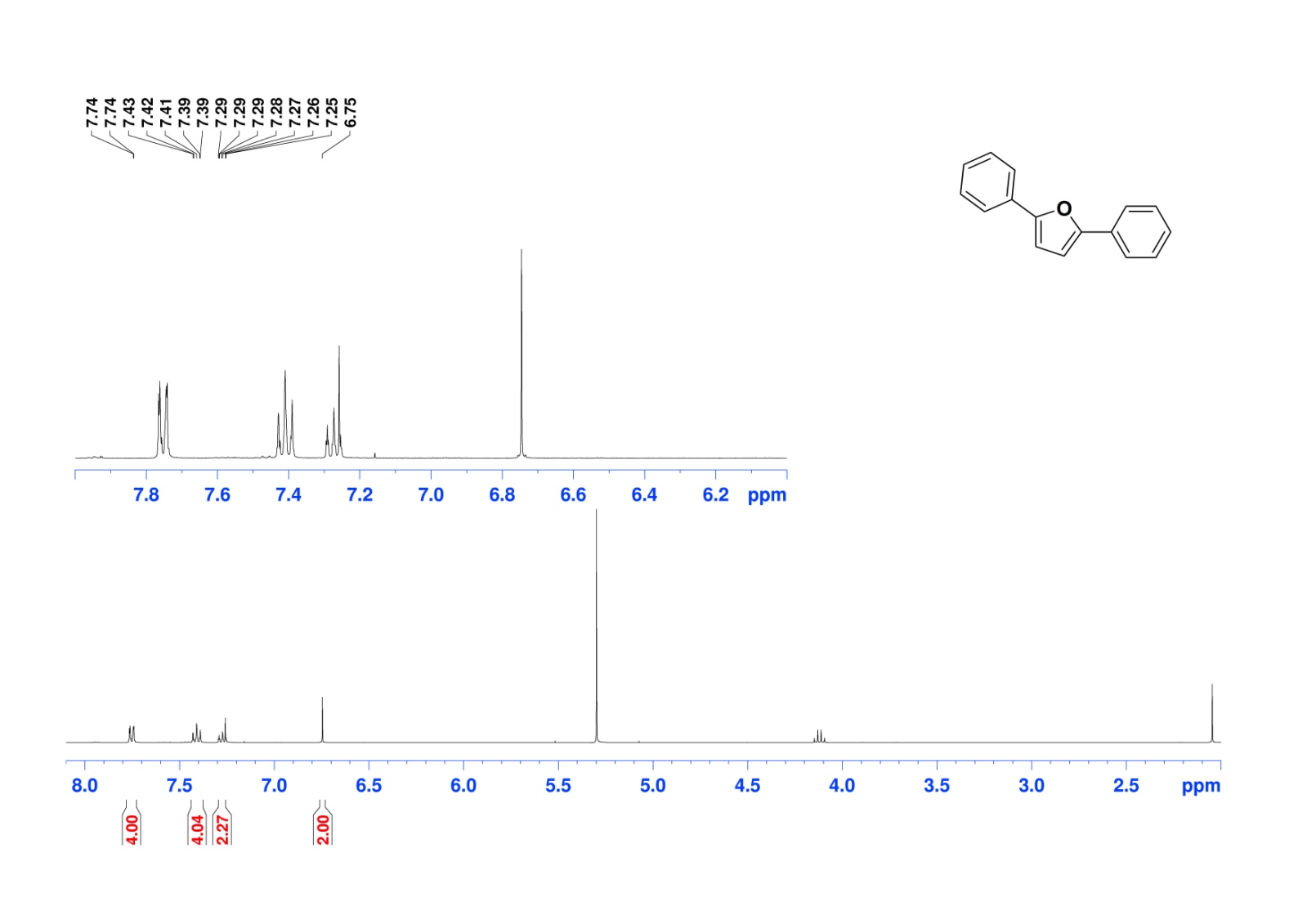

**2,​5-​Diphenylthiophene (4e).** C,N-Pd/OMC (1 mol% of Pd), thiophene (**1e**) (0.2 mmol), diphenyliodonium triflate (**2a**) (0.4 mmol), and deionized water (2.0 ml) were added in a 10 ml vial. The solution was heated with stirring to 60 ºC for 8 hours. **4e** was obtained as a colorless solid (39 mg, 83% yield) after purification by column chromatography (hexanes/ethyl acetate 90/10). *R_f_* = 0.24 (hexanes) **^1^H NMR (400 MHz, CDCl_3_, ppm)** δ 7.64 – 7.63 (d, *J* = 7.8 Hz, 4H). 7.41 – 7.37 (t, *J* = 7.9 Hz, 4H), 7.30 – 7.25 (m, 4H). The ^1^H NMR data were in accordance with literature [25].

**^
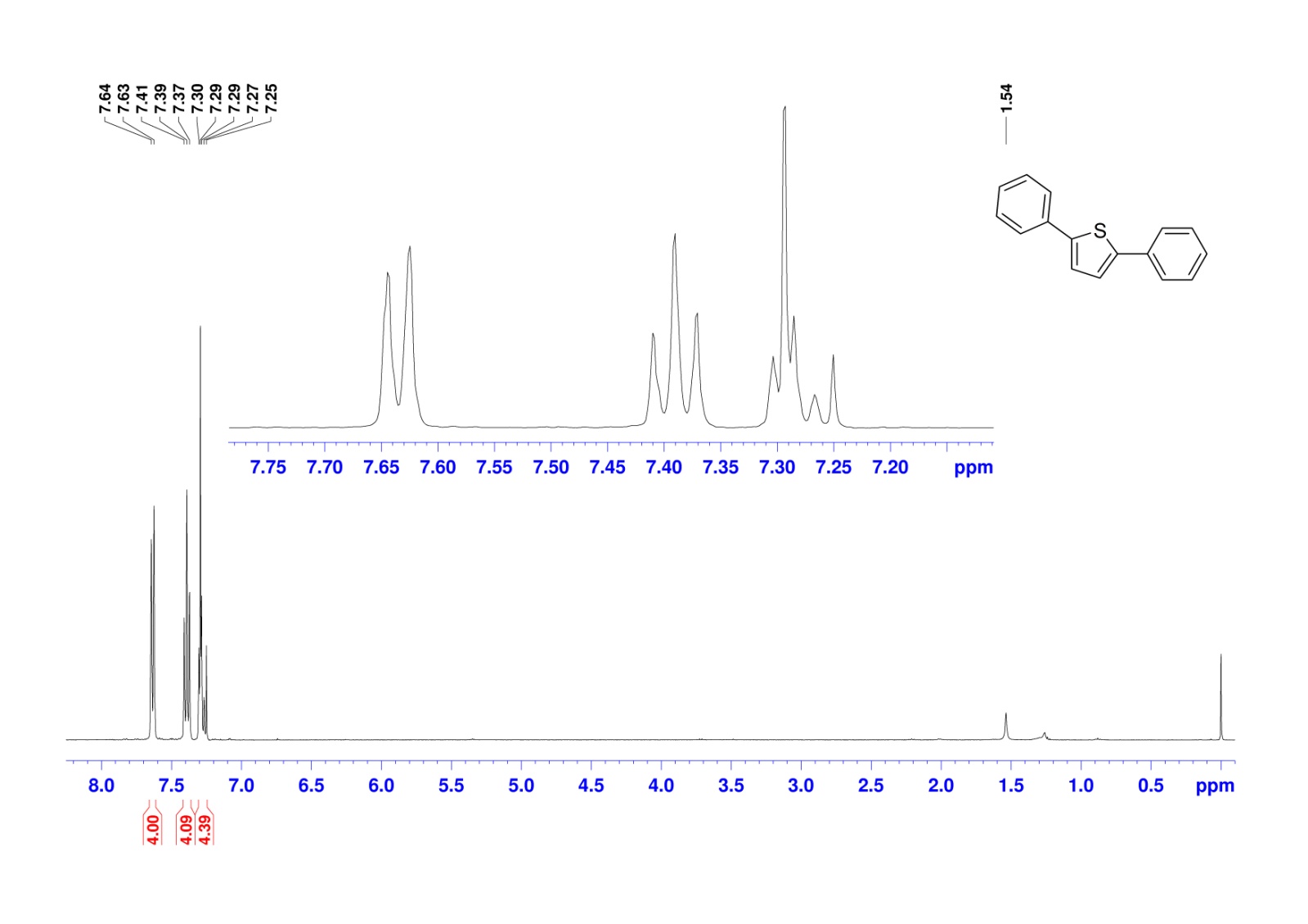
^**

**Supplementary** **References**

1 Madon RJ and Boudart M. Experimental criterion for the absence of artifacts in the measurement of rates of heterogeneous catalytic reactions. *Ind. Eng. Chem. Fund.* 1982; **21**: 438-447.

2 Ehlers P, Petrosyan A and Baumgard J *et al.* Synthesis of 2, 5-Diarylpyrroles by Ligand-Free Palladium-Catalyzed CH Activation of Pyrroles in Ionic Liquids. *ChemCatChem* 2013; **5**: 2504-2511.

3 Deprez NR, Kalyani D and Krause A *et al.* Room Temperature Palladium-Catalyzed 2-Arylation of Indoles. *J. Am. Chem. Soc.* 2006; **128**: 4972-4973.

4 Wilson K, Lee AF and Macquarrie DJ *et al.* Structure and reactivity of sol–gel sulphonic acid silicas. *Appl. Catal., A* 2002; **228**: 127-133.

5 Sakamoto Y, Fukuoka A and Higuchi T *et al.* Synthesis of Platinum Nanowires in Organic− Inorganic Mesoporous Silica Templates by Photoreduction: Formation Mechanism and Isolation. *J. Phys. Chem. B* 2004; **108**: 853-858.

6 Duan LL, Fu R and Zhang BS *et al.* An efficient reusable mesoporous solid-based Pd catalyst for selective C2 arylation of indoles in water. *ACS Catal.* 2016; **6**: 1062–1074.

7 Ravel B and Newville M. ATHENA, ARTEMIS, HEPHAESTUS: data analysis for X-ray absorption spectroscopy using IFEFFIT. *J. Synchrotron. Radi.* 2005; **12**: 537-541.

8 Inoue T, Asakura K and Iwasawa Y. Characterization of Pt/SbOxCatalysts Active for Selective Oxidation of Isobutane by Means of XRD, TEM, and XAFS. *J. Catal.* 1997; **171**: 457-466.

9 Gatla S, Mathon O and Rogalev A *et al.* Influence of Sb on the structure and performance of Pd-based catalysts: an X-ray spectroscopic study. *J. Phys. Chem. C* 2017; **121**: 3854–3861.

10 Rogalev A, Wilhelm F and Jaouen N *et al.* in *Magnetism: A Synchrotron Radiation Approach* 71-93 (Springer Berlin Heidelberg, 2006).

11 Starace AF. Potential-barrier effects in photoabsorption. I. General theory. *Phys. Rev. B* 1972; **5**: 1773.

12 Sham T. L-edge X-ray-absorption spectra of PdAl_3_ and PdCl_2_: A study of charge redistribution in compounds of an element with a nearly full 4d shell. *Phys. Rev. B* 1985; **31**: 1903.

13 Kresse G and Joubert D. From ultrasoft pseudopotentials to the projector augmented-wave method. *Phys. Rev. B* 1999; **59**: 1758.

14 Kresse G and Furthmüller J. Efficient iterative schemes for ab initio total-energy calculations using a plane-wave basis set. *Phys. Rev. B* 1996; **54**: 11169.

15 Perdew JP, Burke K and Ernzerhof M. Generalized gradient approximation made simple. *Phys. Rev. Lett.* 1996; **77**: 3865.

16 Monkhorst HJ and Pack JD. Special points for Brillouin-zone integrations. *Phys. Rev. B* 1976; **13**: 5188.

17 Viñes F, Loschen C and Illas F *et al.* Edge sites as a gate for subsurface carbon in palladium nanoparticles. *J. Catal.* 2009; **266**: 59-63.

18 Yang B, Burch R and Hardacre C *et al.* Influence of surface structures, subsurface carbon and hydrogen, and surface alloying on the activity and selectivity of acetylene hydrogenation on Pd surfaces: A density functional theory study. *J. Catal.* 2013; **305**: 264-276.

19 Chen W, Li D and Peng C *et al.* Mechanistic and kinetic insights into the Pt-Ru synergy during hydrogen generation from ammonia borane over PtRu/CNT nanocatalysts. *J. Catal.* 2017; **356**: 186-196.

20 Teschner D, Novell-Leruth G and Farra R *et al.* In situ surface coverage analysis of RuO_2_-catalysed HCl oxidation reveals the entropic origin of compensation in heterogeneous catalysis. *Nat. Chem.* 2012; **4**: 739.

21 Nadres ET, Lazareva A and Daugulis O. Palladium-catalyzed indole, pyrrole, and furan arylation by aryl chlorides. *J. Org. Chem.* 2010; **76**: 471–483.

22 Cho BS, Bae HJ and Chung YK. Phosphine-free palladium-catalyzed direct bisarylation of pyrroles with aryl iodides on water. *J. Org. Chem.* 2015; **80**: 5302–5307.

23 Hiroya K and Matsumoto S and Ashikawa M *et al.* Cyclization reactions of homopropargyl azide derivatives catalyzed by PtCl4 in ethanol solution: synthesis of functionalized pyrrole derivatives. *Org. Lett.* 2006; **8**: 5349-5352.

24 Schmidt B and Geissler D. Ru-and Pd-Catalysed Synthesis of 2-Arylfurans by One-Flask Heck Arylation/Oxidation. *Eur. J. Org. Chem.* 2011; **2011**: 4814-4822.

25 Do H-Q, Khan RMK and Daugulis O. A general method for copper-catalyzed arylation of arene C− H bonds. *J. Am. Chem. Soc.* 2008; **130**: 15185-15192.
